# Chloride – The Underrated Ion in Nociceptors

**DOI:** 10.3389/fnins.2020.00287

**Published:** 2020-04-08

**Authors:** Bettina U. Wilke, Kai K. Kummer, Michael G. Leitner, Michaela Kress

**Affiliations:** Institute of Physiology, Department of Physiology and Medical Physics, Medical University of Innsbruck, Innsbruck, Austria

**Keywords:** anoctamin 1, glycine receptor, GABA_A_ receptor, NKCC1, KCC2

## Abstract

In contrast to pain processing neurons in the spinal cord, where the importance of chloride conductances is already well established, chloride homeostasis in primary afferent neurons has received less attention. Sensory neurons maintain high intracellular chloride concentrations through balanced activity of Na^+^-K^+^-2Cl^–^ cotransporter 1 (NKCC1) and K^+^-Cl^–^ cotransporter 2 (KCC2). Whereas in other cell types activation of chloride conductances causes hyperpolarization, activation of the same conductances in primary afferent neurons may lead to inhibitory or excitatory depolarization depending on the actual chloride reversal potential and the total amount of chloride efflux during channel or transporter activation. Dorsal root ganglion (DRG) neurons express a multitude of chloride channel types belonging to different channel families, such as ligand-gated, ionotropic γ-aminobutyric acid (GABA) or glycine receptors, Ca^2+^-activated chloride channels of the anoctamin/TMEM16, bestrophin or tweety-homolog family, CLC chloride channels and transporters, *cystic fibrosis transmembrane conductance regulator* (CFTR) as well as volume-regulated anion channels (VRACs). Specific chloride conductances are involved in signal transduction and amplification at the peripheral nerve terminal, contribute to excitability and action potential generation of sensory neurons, or crucially shape synaptic transmission in the spinal dorsal horn. In addition, chloride channels can be modified by a plethora of inflammatory mediators affecting them directly, via protein-protein interaction, or through signaling cascades. Since chloride channels as well as mediators that modulate chloride fluxes are regulated in pain disorders and contribute to nociceptor excitation and sensitization it is timely and important to emphasize their critical role in nociceptive primary afferents in this review.

## Introduction

In the past, excitation of primary afferent neurons has been associated mainly with cation fluxes across the cell membrane, setting the neurons’ excitability, responsiveness to tissue damaging stimuli as well as action potential (AP) generation and propagation. However, the dynamic of Cl^–^ homeostasis and the role of Cl^–^ fluxes across the cell membrane in primary afferents and their spinal connections are gaining increasing attention and turn out to be of critical importance in particular for the development and maintenance of neuropathic pain. In the following review we will summarize which chloride conductances are expressed in DRG neurons and what is currently known about their contribution to nociception and chronic pain development.

## Cl^–^ Ion Homeostasis in DRG Neurons

The intracellular Cl^–^ concentration of neurons is maintained by cell membrane transporters including Na^+^-K^+^-2Cl^–^ cotransporter 1 (NKCC1), or K^+^-Cl^–^ cotransporter 2 (KCC2), the latter becomes prominent in mature neurons (Payne et al., 2003; Ben-Ari et al., 2012; Kaila et al., 2014). Both transporters mediate electroneutral transport of Cl^–^ and utilize the electrochemical gradient generated by the Na^+^-K^+^ ATPase. KCCs in general extrude Cl^–^ from the cell leading to low intracellular Cl^–^ concentration and a negative equilibrium potential for Cl^–^, while NKCCs increase cytoplasmic Cl^–^ concentration by shuffling Cl^–^ into the cell resulting in a more depolarized Cl^–^ equilibrium potential. When the intracellular Cl^–^ concentration is very low and the Cl^–^ equilibrium potential (E_Cl__–_) is below the resting membrane potential (V_*M*_) of the respective neuron, Cl^–^ influx leads to hyperpolarization mediated by net inward flux of negative charge. In contrast, at high intracellular Cl^–^ concentration, activation of a Cl^–^ conductance will depolarize the cell due to Cl^–^ efflux. However, this may still cause inhibition of the respective neuron via two mechanisms: (1) slow membrane depolarization by Cl^–^ efflux causes inactivation of voltage-gated Na^+^ channels that results in reduced excitability and (2) increased Cl^–^ conductance reduces the input resistance of the membrane ‘shunting’ electrical input upon stronger depolarization (Price et al., 2009; Kaila et al., 2014). Strong and rapid depolarization occurs if E_Cl__–_ is near the AP threshold resulting in AP discharge (Prescott et al., 2006; Price et al., 2009).

In contrast to other cell types, sensory neurons, whose cell bodies reside in the DRG, express sustained NKCC1, KCC1 and KCC3 activity but low or even undetectable KCC2 (Rivera et al., 1999; Sung et al., 2000; Kanaka et al., 2001; Coull et al., 2003; Gilbert et al., 2007; Price et al., 2009; Pieraut et al., 2011; Lucas et al., 2012). Because KCC1 and -3 activity is increased by cell swelling but is low under isoosmotic conditions, these cation-chloride cotransporters (CCCs) do not significantly reduce the intracellular Cl^–^ concentration (Gamba, 2005). This is supported by the finding that KCC3 ablation in nociceptors does not affect heat sensitivity (Ding and Delpire, 2014). Therefore, mainly NKCC1 determines the high intracellular Cl^–^ levels in mature DRG neurons in normal isotonic situation. In addition to CCCs, also the Na^+^-independent Cl^–^-HCO_3_^–^-anion exchanger AE3 might contribute to the intracellular Cl^–^ accumulation (Pfeffer et al., 2009). This transporter is expressed in ∼60% of peptidergic and ∼30% of non-peptidergic DRG neurons (Barragan-Iglesias et al., 2014). The actual reversal potential for Cl^–^ in DRG neurons is subject to regulation and varies between −20 mV and −70 mV (Gilbert et al., 2007; Funk et al., 2008). Therefore, activation of Cl^–^ channels usually will cause depolarization, and the degree and velocity of depolarization determines whether this has inhibitory or excitatory consequences (Sung et al., 2000; Prescott et al., 2006; Gilbert et al., 2007; Funk et al., 2008). Furthermore, localized signaling and intracellular Cl^–^ diffusion additionally increase heterogeneity and complexity of anion currents (Kuner and Augustine, 2000; Gulledge and Stuart, 2003; Doyon et al., 2011; Raimondo et al., 2012). Thus, depending on spatial and temporal fluctuations of Cl^–^ levels, Cl^–^ ion channel activation can have different effects on the overall activity of primary afferents.

CCCs, like NKCC, KCC and the Na^+^-Cl^–^-cotransporter NCC, and anion transporters such as the Na^+^-dependent Cl^–^-2HCO_3_^–^-exchanger and the Na^+^-independent Cl^–^-HCO_3_^–^-anion exchanger, link different ion species including H^+^ and HCO_3_^–^ and even the membrane potential to Cl^–^ level regulation (Doyon et al., 2011, 2016). In addition, Cl^–^ channels are permeable to other anions, of which HCO_3_^–^ is physiologically most important since sustained HCO_3_^–^ outward flux through anion channels is ensured by free diffusion of CO_2_ across the membrane (Price et al., 2009). This allows for constant replenishment of HCO_3_^–^ catalyzed by carbonic anhydrase which is detectable in ∼30% of DRG neurons (Prabhakar and Lawson, 1995; Price et al., 2009). The contribution of HCO_3_^–^ to the total anion current through the open anion channels can change when the driving force for Cl^–^ is altered due to a reduction in Cl^–^ gradient (Cordero-Erausquin et al., 2005). A collapse of the Cl^–^ gradient can lead to an increased proportion of HCO_3_^–^ to the total anion current or even to a biphasic anion current response. The E_Cl__–_ can change rapidly, particularly during strong activation and in small compartments, or on a long time range during pain conditions due to regulation of CCCs (Coull et al., 2003; Funk et al., 2008; Wright et al., 2011; Doyon et al., 2016; Li et al., 2016).

As a consequence of the high intracellular Cl^–^ concentration mediated by the active accumulation of Cl^–^ by NKCC1, for example GABA-evoked depolarizing currents are observed in primary afferents at resting membrane potential (Sung et al., 2000); likewise, activation of G protein-coupled receptors, e.g. by lysophosphatidic acid (LPA) and sphingosine-1-phosphate (S1P), has been shown to activate excitatory chloride conductances (Ponsioen et al., 2009; Camprubi-Robles et al., 2013; Qi et al., 2018).

In contrast to the well-established contribution of deregulated CCCs with disturbed Cl^–^ homeostasis and rising Cl^–^ levels in spinal cord neurons to the pathophysiology of pain disorders (Coull et al., 2003), the regulation of CCCs in primary sensory neurons in inflammation and pain is still controversially discussed. In an arthritis model, NKCC1 is downregulated, whereas sciatic nerve injury and inflammatory mediators increase NKCC1 expression and activity (Morales-Aza et al., 2004; Funk et al., 2008; Chen et al., 2014; Modol et al., 2014). At the same time, these mediators reduce expression of KCC2 in DRGs, which leads together with NKCC1 activation to increased intracellular Cl^–^ levels and nociceptor excitability (Funk et al., 2008; Pieraut et al., 2011). Thus, persistent inflammation can increase GABA-induced depolarization by affecting Cl^–^ homeostasis in DRG neurons (Zhu et al., 2012), while ablation of NKCC1 or pharmacological inhibition by bumetanide increases the latency in the hot plate or tail flick tests and alleviates thermal and mechanical hypersensitivity after sciatic nerve lesion (Modol et al., 2014). Similarly, the Cl^–^-HCO_3_^–^ exchanger AE3 is upregulated in a formalin-induced pain model and a Cl^–^-HCO_3_^–^ anion exchange inhibitor blocks the evoked allodynia and hyperalgesia (Barragan-Iglesias et al., 2014). These reports highlight the significance of Cl^–^ homeostasis and Cl^–^ conductances in nociceptors.

## Chloride Channels/Transporters Expressed in Primary Sensory Afferents

**FIGURE 1:**
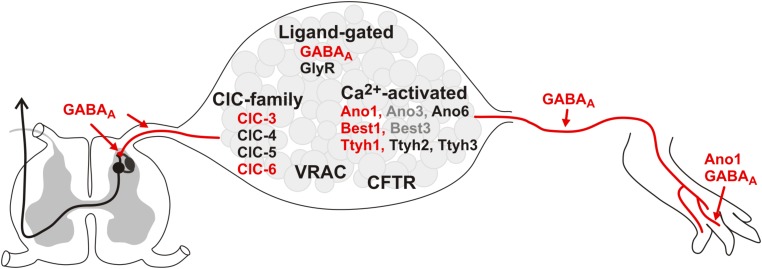
Cl^–^ conductances expressed in DRGs. Expression of the depicted Cl^–^ channels/transporters has been demonstrated in DRGs. The channels in red have been associated with pain: Their activity in nociceptors affects pain sensation and/or their expression is modulated in primary afferents in pain conditions. In contrast, for the conductances written in black neither a clear contribution to pain behavior, nor a regulation in pain models has been demonstrated so far. The gray candidates show low expression and no particular role in nociceptors has been assigned to them yet. As most data exists on the mRNA/protein level within the DRG, little is known about the distribution of the Cl^–^ conductances within the central or peripheral axons and their terminals. So far, only Ano1 and GABA_A_ have been functionally established at the peripheral axon terminal, while GABA_A_ is also involved in presynaptic inhibition of the primary afferents in the dorsal horn of the spinal cord.

Cl^–^ conductances in primary afferents can be carried by different members of the chloride channel superfamily (Figure 1) and are of importance at the peripheral nerve terminal in the target tissue, along the peripheral axon, on the cell soma, as well as at synaptic terminals within the spinal dorsal horn. However, neuromorphological evidence regarding the subcellular localization of the chloride channels is sparse, as most evidence is derived from the cell body of DRG neurons. Besides the pentameric ligand-gated ionotropic GABA_A_ and glycine receptors, the large class of Cl^–^ channels and transporters comprises several structurally unrelated families. These include Ca^2+^-activated Cl^–^ channels of the anoctamin (Ano or TMEM16), bestrophin (Best) and tweety homolog (Ttyh) families, the CLC channel/transporter family, the *cystic fibrosis transmembrane conductance regulator* (CFTR), volume-regulated anion channels (VRAC) that are formed by LRRC8 proteins, and SLCO2A1 as the molecular correlate of maxi-anion channels. Of those, GABA_A_, Ano1, Best1, Thyh1, CLC-3 and CLC-6 have been associated with nociception: either their expression is altered in pain states or their activity modulates pain (see Figure 1, marked in red), however other candidates may also contribute and can be addressed by an increasing number of genetic, chemogenetic, optogenetic and pharmacological tools (Bormann, 2000; Enna and McCarson, 2006; Poet et al., 2006; Boudes et al., 2009; Zeilhofer et al., 2009; Liu et al., 2010; Cho et al., 2012).

### Ligand-Gated Chloride Channels

#### GABA_A_ Receptors

γ-aminobutyric acid (GABA) is the main inhibitory neurotransmitter of the central nervous system (Olsen, 2002). It binds to two different receptor types, the ionotropic GABA_A_ receptors and the metabotropic Gi/o protein-coupled GABA_*B*_ receptors. GABA_A_ receptors are members of the Cys-loop receptor family of ligand-gated ion channels that share a pentameric structure with a large N-terminal extracellular domain for ligand binding, four transmembrane regions including the pore-forming segment, and one large cytoplasmic loop for intracellular modifications (Galaz et al., 2015). To date 19 different subunits have been identified (six α-, three β-, three γ-, three ρ- and one of each ε-, δ-, θ-, and π-subunits) (Sigel and Steinmann, 2012). The major GABA_A_ channel isoform in adult DRG neurons is composed of two α1- and β2-subunits and one γ2-subunit (Sigel and Steinmann, 2012). Alternative subunit assemblies define different functional and physiological properties.

GABA has been implicated as an important modulator at different levels of the pain pathway, although mainly micro-circuitries within the spinal dorsal horn (SDH) and supraspinal brain regions have been investigated (Enna and McCarson, 2006). Already in the 1970s it was established that primary sensory neurons respond to GABA stimulation with depolarization (De Groat et al., 1972). Since then, a multitude of studies link this enigmatic response to a possible chloride conductance (Desarmenien et al., 1979, 1980, 1981; Gallagher et al., 1983a, b). In embryonic DRG neurons, GABA generates an inward current, which is inhibited by the GABA_A_ antagonists bicuculline, picrotoxin and TBPS (Valeyev et al., 1999). The attributes of these GABA-induced currents depend on the primary afferent cell type, with TTX-sensitive and capsaicin-insensitive neurons generating larger currents than capsaicin-sensitive nociceptors (White, 1990). Accordingly, unique developmental expression patterns of different GABA_A_ subunits are reported: α2 and β3 subunit mRNA is expressed in all embryonic and adult DRG neurons, while β2 mRNA is only present in adult ones; 37% of DRG neurons express the GABA_A_ γ- subunit (Furuyama et al., 1992; Maddox et al., 2004); GABA_A_ subunits α1, α6, β1, γ2L, and ρ2 are absent in DRG, and the δ-subunit is only weakly expressed (Ma et al., 1993; Maddox et al., 2004; Du et al., 2017).

By now, a role for GABA_A_-mediated currents has been established in all morpho-functional nociceptor compartments, where they have different roles in pain regulation and distinct GABA sources: GABA released at an injury site sensitizes nociceptors at the peripheral terminal, while GABA acting in a paracrine mode on the cell bodies within the DRG has mostly antinociceptive effects (Du et al., 2017). GABA_A_ responses can be evoked both in the dorsal root as well as in the sciatic nerve, demonstrating the presence of these receptors on the central and peripheral axon (Bhisitkul et al., 1987). GABA_A_ receptors located at central terminals of primary afferents in the spinal dorsal horn respond to GABA-release from spinal interneurons and induce presynaptic inhibition of synaptic inputs, which in most sensory systems is responsible for contrast enhancement and gain control (Zimmerman et al., 2019). Activation of these GABA_A_ receptors by GABA released at axo-axonic synapses induces Cl^–^ efflux that causes primary afferent depolarization. Paradoxically, this depolarization leads to a reduced transmitter release from afferent terminals by either shunting inhibition, inactivation of voltage-gated Na^+^ channels or inactivation of voltage-gated Ca^2+^ channels and is thus antinociceptive (Rudomin and Schmidt, 1999; Kullmann et al., 2005; Lidierth, 2006; Witschi et al., 2011). Sensory afferents synapse to different dorsal horn inhibitory GABA interneurons that are further modulated by descending projections from cortex and brainstem. These interneurons then affect the transmission of painful signals from the nociceptor to the secondary neuron. Loss of presynaptic, GABA_A_-mediated inhibition leads to tactile hypersensitivity and impaired texture discrimination (Zimmerman et al., 2019). The descending projections can set pain thresholds based on internal and emotional states, with acute stress and expected pain producing analgesia, while chronic stress and anxiety facilitate pain (Porreca et al., 2002; Basbaum et al., 2009; Jennings et al., 2014; Francois et al., 2017). The main source for descending pain modulation is the rostroventral medulla (RVM), which harbors both glutamatergic ON neurons that facilitate nociception by excitation of primary afferent terminals and/or excitatory neurons within SDH (Heinricher et al., 2009) and antinociceptive OFF cells that provide inputs onto nociceptive primary afferents and thus suppress pain transmission (Budai and Fields, 1998). Interestingly, the majority of RVM-derived descending input is GABAergic, with indirect GABAergic projections via SDH GABAergic interneurons that, when inhibited, lead to mechanical hyposensitivity (Francois et al., 2017), as well as GABAergic inputs to sensory afferents that, when inhibited, increase both heat and mechanical sensitivity (Zhang et al., 2015).

In inflamed tissue, blood and immune cells actively release GABA, which may directly act on primary afferent nerve terminals and induce excitation and sensitization (Bhat et al., 2010; Bravo-Hernandez et al., 2014). The GABA_A_ subunits α1 and β2/3 have been detected by immunohistochemistry in 10–14% of unmyelinated peripheral axons in the cat glabrous skin (Carlton et al., 1999). Peripheral administration of the selective GABA_A_ receptor agonist muscimol evokes nocifensive behavior whereas the GABA_A_ blockers picrotoxin or bicuculline inhibit formalin-induced pain-like behavior (Bravo-Hernandez et al., 2014; Jang et al., 2017). GABA-activated currents are modified by inflammatory mediators, like prostaglandin E2 (PGE_2_), bradykinin, histamine, ATP or interferon gamma (Sokolova et al., 2001; Vlachova et al., 2001; Labrakakis et al., 2003; Vikman et al., 2003; Toulme et al., 2007). The neurotransmitter serotonin (5-HT) or a 5-HT_2_ receptor agonist potentiate, while dopamine D1 receptor agonists, *N*-methyl-D-aspartate (NMDA) – but not kainic acid – as well as adenosine and caffeine inhibit GABA currents in DRG neurons (Xi and Akasu, 1996; Hu and Li, 1997; Li et al., 2004). Likewise, neuropeptides such as substance P, neurokinin A, and neurokinin B inhibit GABA_A_-induced currents, likely mediated by protein kinase C, but not protein kinase A (Guan et al., 1994; Wu et al., 1994; Yamada and Akasu, 1996; Akasu and Yamada, 1997; Yang et al., 2003; Si et al., 2004; Li L. et al., 2015).

Thus, sustained inflammation or neuropathic alterations appear to strongly affect the function and expression of GABA currents and receptors. Twenty-four hours after complete Freund’s adjuvant (CFA)-induced inflammation, retrogradely-labeled DRG neurons innervating the inflamed knee joint show increased GABA sensitivity and a decreased AP threshold (Chakrabarti et al., 2018). Following nerve ligation or chronic constriction injury, GABA-induced conductances and depolarization in small, medium, and large DRG neurons are attenuated (Bhisitkul et al., 1990; Chen et al., 2014; Ran et al., 2014). This is accompanied by respective downregulation of the GABA_A_ α2-subunit by 30% in ipsilateral DRGs and reduction of the number of γ2-subunit mRNA expressing neurons (Obata et al., 2003; Obradovic et al., 2015). Consequently, injections of the GABA agonists muscimol or gaboxadol into the DRG immediately after nerve injury attenuate, whereas the GABA antagonists bicuculline or picrotoxin aggravate pain-like behavior (Naik et al., 2008, 2012; Ran et al., 2014). Upregulation of endogenous GABA within the sensory ganglion via GABA uptake inhibition alleviates thermal hyperalgesia, whereas knockdown of the α2 subunit further decreases pain thresholds (Obradovic et al., 2015). In contrast to the reduction of GABA currents described so far, GABA-induced conductances increase in axotomized cutaneous neurons and this is attenuated by BDNF, whereas NGF has no effect (Oyelese and Kocsis, 1996; Oyelese et al., 1997). DRG neuron cultures, which to a certain extent represent an axotomy model *per se*, also develop increased GABA_A_ current densities with time in culture (Lee et al., 2012). Other pain models such as formalin or reserpine injections are associated with upregulated α5 mRNA and protein expression in DRGs and spinal cord, and peripheral or intrathecal administration of an α5 antagonist prevents and reverses mechanical hypersensitivity (Bravo-Hernandez et al., 2016; De la Luz-Cuellar et al., 2019). Albeit there is an increasing body of data, the contribution of GABA_A_ receptors in nociceptors to pathological pain is still controversial.

#### Glycine Receptors

The ligand-gated ionotropic glycine receptor (GlyR) is activated by amino acid ligands, with glycine, taurine and beta-alanine being the most common agonists, whereas the alkaloid strychnine acts as a high affinity antagonist (for reviews see Lynch, 2009; Dutertre et al., 2012; Galaz et al., 2015). Four different types of alpha subunits (GlyR α1-4) and one beta subunit (GlyR β) form heteropentameric ion channels in a 2α:3β stoichiometry (Dutertre et al., 2012). Homomeric receptors composed solely of α subunits have been observed in recombinant expression systems whereas β subunits are retained in the endoplasmic reticulum and are thus unable to form functional channels (Dutertre et al., 2012; Galaz et al., 2015).

The role of glycine receptors in pain modulation mainly relates to the SDH, where α3β heteromeric GlyRs are important anion channels of glycinergic inhibitory neurotransmission in the superficial SDH (Lynch, 2009). α3 subunits are mainly clustered by gephyrin at postsynaptic membranes in lamina 2, and colocalize with α1 subunits in around half the cases (Harvey et al., 2004). These α3 containing GlyRs are phosphorylated by proteinkinase A activated downstream of PGE_2_ receptor EP_2_, thereby diminishing glycinergic inhibitory input to lamina 2 neurons (Ahmadi et al., 2002; Harvey et al., 2004). In GlyR α3^–/–^ mice, this PGE_2_-mediated reduction of inhibitory postsynaptic potentials of lamina 2 neurons is abolished, together with alleviated pain sensitization in response to chronic peripheral inflammation, while acute inflammatory pain stimuli are not affected (Harvey et al., 2004). In addition, analgesic effects of cannabinoids in different models of chronic inflammatory and neuropathic pain are absent in these mice (Xiong et al., 2012). Tissue damage during the neonatal period decreases GlyR-mediated input onto SDH GABAergic and glutamatergic neurons in adulthood and both mRNA and protein levels of the GlyR β subunit are upregulated in spinal cord in animals subjected to prolonged pain (Li et al., 2013; Galaz et al., 2015). Thus a major role for GlyRs in SDH circuits is well documented whereas the reports on glycinergic effects on primary afferents are sparse and inconsistent. In chicken embryo DRGs, uptake of [14C]2-deoxyglucose as a marker of excitatory activity is facilitated in response to glycine stimulation, and taurine – a GlyR (but also GABA_A_) agonist - causes depolarizing responses in frog primary afferents (Saji and Obata, 1981; Padjen et al., 1989). In contrast, GlyRs are not involved in presynaptic modulation of transmitter release in rat spinal cord sections and neither taurine nor glycine show any detectable agonist activity in mammalian DRG neurons (Robertson, 1989; Valeyev et al., 1996, 1999; Betelli et al., 2015). Forty four percent of all rat DRG neurons express GlyR β subunit mRNA (Furuyama et al., 1992), L5 DRG neurons express both α3 and α1 GlyR subunits, and protein expression is decreased after intrathecal PGE_2_ injection (Wang et al., 2018). DRG and SDH neuron co-cultures develop inhibitory glycinergic synapses which are able to generate inhibitory synaptic transmission at DRG neurons (Shypshyna and Veselovs’kyi, 2010). This suggests that GlyR in primary afferent neurons are predominantly involved in setting transmission efficacy at SDH synapses but are not involved in nociceptive transduction.

### Calcium-Activated Chloride Channels

#### Anoctamins

**FIGURE 2:**
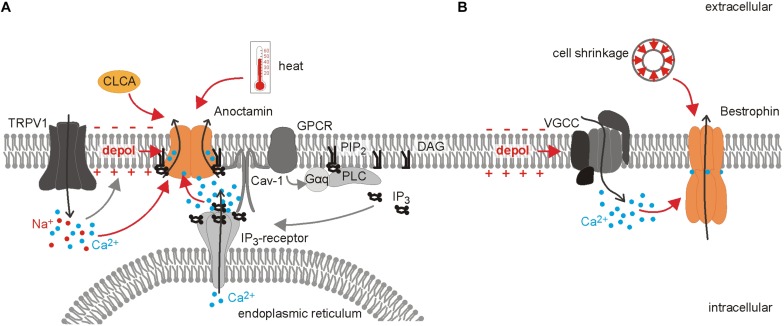
Differential activation of Ca^2+^-gated Cl^–^ channels in nociceptors. Overview of the distinct regulation of the Ca^2+^-gated Cl^–^ channels anoctamin and bestrophin. **(A)** Ano1 is found in a signaling complex together with GPCR, Caveolin-1 (Cav-1) and the Inositol-1,4,5 trisphosphate (IP_3_) receptor in the membrane of the endoplasmic reticulum. This proximity of signaling molecules allows for a localized Ca^2+^ rise which is sufficient to activate the Cl^–^ channel Ano1, while the Ca^2+^ increase mediated by VGCCs does not affect anoctamin currents. Binding of phosphatidylinositol -4,5-bisphosphate (PIP_2_) to anoctamin is necessary for channel opening under low Ca^2+^ concentration and prevents channel desensitization when Ca^2+^ concentration is high. Ano1 is further activated by heat and membrane depolarization. Direct interaction of TRPV1 and Ano1 leads to subsequent activation of anoctamin mediated by the TRPV1-induced depolarization and Ca^2+^ influx. Ano1 and TRPV1 act as peripheral heat sensors, but whether the full signaling complex exists at the peripheral nerve terminals has not been unequivocally shown so far. Additionally, the soluble N-terminus of chloride channel accessory (CLCA) increases surface availability of anoctamin. **(B)** In contrast to anoctamin, bestrophins can be activated by the Ca^2+^ influx through VGCCs and cell shrinkage.

Ano1 was identified in 2008 as the first member of the anoctamin protein family (Ano; TMEM16) (Caputo et al., 2008; Schroeder et al., 2008; Yang et al., 2008). This protein family consists of ten members, Ano1-10, and the recent structural data revealed ten transmembrane domains (Paulino et al., 2017). The subunits assemble as dimers with two distinct pores to serve divers functions ranging from calcium level regulation to lipid scramblase and ion channel activity (Caputo et al., 2008; Schroeder et al., 2008; Yang et al., 2008; Pifferi et al., 2009; Wanitchakool et al., 2017). Alternative splicing further increases the number of isoforms with different biophysical properties and expression (Ferrera et al., 2009; Mazzone et al., 2011; Ertongur-Fauth et al., 2014). Ano1, 2, and 6 have been shown to convey Ca^2+^-activated Cl^–^ conductances, although Ano6 can conduct both anions and cations depending on intracellular Ca^2+^ concentration and membrane potential (Caputo et al., 2008; Schroeder et al., 2008; Yang et al., 2008; Pifferi et al., 2009; Ye et al., 2019). Ano conductances are synergistically regulated by intracellular Ca^2+^, membrane depolarization, membrane lipids and heat (Cho et al., 2012; Schreiber et al., 2018; Le et al., 2019; Lin et al., 2019). The Ca^2+^ sensitivity represents a hallmark of Ano function: Cryo-EM structures of mouse Ano1 reveal two Ca^2+^ binding sites within the inner vestibule of the hour-glass shaped pore (Paulino et al., 2017). Ca^2+^ binding leads to conformational changes particularly in the α6 transmembrane domain rendering the pore conductive (Paulino et al., 2017). The Ca^2+^ sensitivity is dependent on membrane voltage and temperature and varies strongly between the different splice variants ranging from approximately 100 to 400 nM for Ano1 and from <1 μM up to 100 μM for Ano6 (Ferrera et al., 2009; Grubb et al., 2013; Ertongur-Fauth et al., 2014; Strege et al., 2015; Lin et al., 2019). In native cells, the organization of Ano1 in microdomains/signaling complexes together with caveolin-1 allows for very specific signaling downstream of Ca^2+^, despite the various sources of this ion, like TRP channels, voltage-gated calcium channels (VGCCs), Inositol-1,4,5-trisphosphate (IP_3_)-receptors on the endoplasmic reticulum, or store-operated calcium entry (Jin et al., 2013, 2016). In nociceptors, Ano1 is specifically activated by local Ca^2+^ signals mediated by TRPV1 and inflammatory mediators like serotonin or bradykinin via G-protein coupled receptors (GPCRs) activating the IP_3_-receptor cascade (Liu et al., 2010; Jin et al., 2013; Salzer et al., 2016) (see Figure 2). This activation of Ano1 has not only been demonstrated at the soma by electrophysiological recordings, but was also shown in behavioral tests, confirming its functional importance at the peripheral nerve ending (Liu et al., 2010). Ano1 channels localize to microdomains in the membrane and interact with the GPCR and IP_3_R (Jin et al., 2013). This compartmentalization is lipid raft-dependent and might (1) allow for a sufficient local Ca^2+^ concentration for channel activation and (2) shield Ano1 from global Ca^2+^ waves (Jin et al., 2013, 2016). This may impede the direct translation of results obtained from expression systems to native cells and vice versa. Furthermore, the loop between transmembrane domain 2 and 3, which is involved in forming the bradykinin/IP_3_-receptor signaling unit, contains two differentially-spliced segments (Ferrera et al., 2009; Jin et al., 2013). In nociceptors neither the expression pattern of the splice variants nor the potential regulation of splicing in (chronic) pain states have been analyzed, but might affect the interaction of the anoctamins with other proteins as well as their Ca^2+^-sensitivity and thus bear an additional mode of channel regulation. Anoctamin channel activity also responds to changes in the membrane environment: upon heterologous expression, inhibition of phospholipase A_2_ suppresses human Ano1- and Ano6-mediated currents (Schreiber et al., 2018). Furthermore, binding of the phospholipid phosphatidylinositol-4,5-bisphosphate (PIP_2_) is necessary for Ano1 channel activity under submaximal Ca^2+^ conditions, while it prevents fast channel desensitization under Ca^2+^ saturation (Le et al., 2019). Activation of Gq-PCR could thus activate Ano1 via Ca^2+^-release from the endoplasmic reticulum by IP_3_, but would counteract Ano1 activation due to PIP_2_ depletion (Le et al., 2019). This is of particular interest in chronic pain states, because PIP_2_/Ca^2+^-signaling is altered under these conditions demonstrated by elevated IP_3_-levels in chronic constriction injury of the sciatic nerve (Zhang et al., 2018).

This complex activation points to two roles of Ano channels in nociceptors: Firstly, they serve as primary sensory channels to detect noxious heat (Cho et al., 2012), and secondly, they are involved in the amplification of intracellular signals affecting membrane voltage (Jin et al., 2013). mRNA of all three anoctamins is expressed in mouse and human trigeminal and dorsal root ganglia (Boudes et al., 2009; Flegel et al., 2015). Ano1 is found primarily in small-diameter nociceptors and upregulated in formalin and neuropathic pain models and associated with increased excitability of sensory neurons (Yang et al., 2008; Liu et al., 2010; Cho et al., 2012; Jin et al., 2013; García et al., 2014; Deba and Bessac, 2015; Pineda-Farias et al., 2015; Takayama et al., 2015; Zhang et al., 2018; Chen et al., 2019). Depletion of Ano1 increases the response latency of mice to noxious heat stimuli and this stresses the importance of Ano1 as a physiological heat sensor (Cho et al., 2012). However, Ano1 does not only complement the role of TRPV1 due to their redundant function as a primary temperature sensor, but also associates with TRPV1 channels and thus amplifies TRPV1-mediated currents in expression systems and DRG neurons (Takayama et al., 2015) (see Figure 2). This coupling between TRPV1 and Ano1 also seems to be relevant *in vivo*: Activation of Ano1 induces nocifensive behavior in mice, although this effect could be largely due to direct TRPV1 activation (Deba and Bessac, 2015; Liu et al., 2016). Pharmacological inhibition or conditional knockout of Ano1 alleviates thermal and mechanical hyperalgesia both in neuropathic and inflammatory pain models (Lee et al., 2014; Pineda-Farias et al., 2015; Chen et al., 2019).

#### Bestrophins

Four members of the Bestrophin family, Best1-4, have been identified in humans and three paralogs are found in mice (Hartzell et al., 2008). The X-ray structure of the chicken and bacterial bestrophin paralog has revealed that the subunits assemble as homo- or heteropentamers forming a central pore, which can conduct anions as large as glutamate and GABA (Sun et al., 2002; O’Driscoll et al., 2008, 2009; Park et al., 2013; Bharill et al., 2014; Kane Dickson et al., 2014; Yang et al., 2014). Both cell volume as well as intracellular Ca^2+^ regulate the largely voltage-independent bestrophin currents: cell shrinkage by hyperosmotic solution inhibits hBest1 and mBest2 by 50–70%, while cell swelling causes a smaller and less robust current increase (Fischmeister and Hartzell, 2005). The channel displays a high Ca^2+^ sensitivity (EC_50_ ∼150–200 nM), which renders it partially conductive under resting Ca^2+^ conditions, and can be activated by VGCCs (Hartzell et al., 2008; O’Driscoll et al., 2008; Boudes et al., 2009; Lee et al., 2010). As for anoctamins, the Ca^2+^ sensitivity of bestrophins is attributable to direct interaction of the divalent cation with an acidic amino acid cluster below the membrane-cytosol interface found in each subunit (Kane Dickson et al., 2014; Paulino et al., 2017). This Ca^2+^-clasp is formed by the proximal C-terminus and the N-terminus of the adjacent subunit (Kane Dickson et al., 2014). Interestingly, a splice variant of Best1 lacking the conserved N-terminal domain, including the first transmembrane domain, still produces a Cl^–^ conductance that is activated by Ca^2+^ when heterologously expressed (Kuo et al., 2014). Just as for Best1, several splice variants are also known for Best3 (Golubinskaya et al., 2019). The role of bestrophins in nociception has not been fully established yet, but both Best1 and Best3 mRNA are detected in mouse DRGs and Best1 mRNA and protein expression is shown in rat DRGs (Al-Jumaily et al., 2007; Boudes et al., 2009; Pineda-Farias et al., 2015). Expression of bestrophin and the corresponding conductance seems to be limited to medium-sized DRG neurons (André et al., 2003). Upregulation of Best1 in mice after sciatic nerve transection is compensated for in Best1 knockout animals by upregulation of Best3 (Boudes et al., 2009). Although an increase in Best1 expression is not observed after spinal nerve ligation in rats, intrathecal injection of Best1 antibody reduces Best1 protein in DRG and spinal cord and attenuates spinal nerve ligation-induced tactile allodynia, indicating a contribution of bestrophin to neuropathic pain (Pineda-Farias et al., 2015). In addition to the regulation of bestrophins themselves, these channels may be affected by altered Ca^2+^ signaling in nociceptors, for example as a consequence of VGCC upregulation in inflammation (Bourinet and Zamponi, 2005).

#### Tweety-Homolog

Three genes, Ttyh1-3, form the Ca^2+^-activated Tweety-homolog Cl^–^ channel family and strong mRNA expression of all three members is detected in mouse and human trigeminal ganglia and DRG (Flegel et al., 2015). Each member is sufficient to produce a swelling-induced volume-regulated Cl^–^ conductance (VRAC_Cl, swell_) with very similar properties to native VRAC_Cl, swell_ which is predominantly found in astrocytes (Han et al., 2019). However, Ttyh2 and Ttyh3 have originally been introduced as Ca^2+^-sensitive channels with a large single-channel conductance, while only the C-terminally spliced Ttyh1 is activated in a Ca^2+^-independent fashion by hypertonic solution (Suzuki and Mizuno, 2004). The outwardly rectifying native VRAC_Cl, swell_ current with significant glutamate permeability is independent of Ca^2+^, but sensitive to inhibitors for tyrosine kinase and mitogen-activated protein kinase (Han et al., 2019). Therefore, the assignment of tweety homologs to Ca^2+^-activated, volume-activated or even Maxi-chloride conductances is inconsistent and needs further investigation. Of note, Ttyh1 is downregulated in DRGs after sciatic nerve transection (Al-Jumaily et al., 2007; Boudes et al., 2009; Flegel et al., 2015). However, since RNA-sequencing of mouse cerebral cortex revealed stronger expression of Ttyh1 and 3 in astrocytes compared to neurons, while expression of Ttyh2 in neurons is very low but rather appears in oligodendrocytes, it can be assumed that the high expression in DRGs is attributable to a function in non-neuronal cells rather than a relevant anion conductance in nociceptors (Zhang et al., 2014).

#### Chloride Channel Accessory (ClCA)

Chloride channel accessory has previously been described as another distinct family of Ca^2+^-activated Cl^–^ channels with four orthologs identified in humans and eight orthologs in mice, of which one might be a pseudogene (Gandhi et al., 1998; Gruber et al., 1998, 1999; Evans et al., 2004; Loewen and Forsyth, 2005; Patel et al., 2009). Yet, based on bioinformatic analysis, further studies revealed that ClCA genes encode soluble proteins with or without a transmembrane domain or GPI anchor that do not yield a Cl^–^ conductance *per se*, but rather modulate other Cl^–^ channels (Gibson et al., 2005; Mundhenk et al., 2006; Patel et al., 2009). Secreted ClCA1 enhances Ano1 surface expression which in turn leads to increased current density (Sala-Rabanal et al., 2015) (see Figure 2). mRNA transcripts of ClCA1, 2, 3, and 5 are expressed in mouse DRGs, and ClCA5 is downregulated after axotomy (Al-Jumaily et al., 2007; Imhof et al., 2011). In contrast, a 1724-fold increase of ClCA3 mRNA transcripts is found after induction of inflammatory pain in an antigen-induced model for arthritis, but a role for this protein could so far not be corroborated in a follow-up study, where ClCA3 knockout mice show a minor reduction in joint swelling but no pain phenotype (Ebbinghaus et al., 2014). Nonetheless, CICAs may be functional regulators of nociception due to their paracrine modulation of other Cl^–^ channels in neighboring neurons in the DRG and thus deserve to be further addressed.

### CLC Family of Chloride Channels and Transporters

Jentsch et al. (1990) cloned the first voltage-gated Cl^–^ channel from *Torpedo*, CLC-0, as the founding member of the CLC family and only one year later they identified and characterized the first mammalian homolog from rat skeletal muscle (Steinmeyer et al., 1991). To date, the mammalian CLC family comprises nine members that can be subdivided into plasma membrane Cl^–^ channels (CLC-1, CLC-2, CLC-Ka, and CLC-Kb) and secondary active 2Cl^–^/H^+^ exchangers (CLC-3, CLC-4, CLC-5, CLC-6, and CLC-7). They are generally located within intracellular (lysosomal and endosomal) membranes. CLC family members have 18 α-helices located within the membrane and share homodimeric co-assembly into functional channels/transporters, with one ion conduction path per subunit (Jentsch and Pusch, 2018). Some members (CLC-2, CLC-3, CLC-5, and CLC-7) are widely expressed whereas others exhibit strict tissue-specific expression (Jentsch et al., 2005; Jentsch and Pusch, 2018). Lumbar DRGs of mice express mRNA transcripts encoding CLC-3, CLC-4, CLC-5, and CLC-6 (Qi et al., 2018) and protein expression in DRGs is demonstrated for CLC-3 and CLC-6 (Poet et al., 2006; Pang et al., 2016). Only few studies address the importance of CLC proteins in the peripheral nervous system, but these demonstrate that CLC proteins are utterly important for determining excitability of DRG neurons under (patho)physiological conditions.

#### CLC-3 and CLC-5

CLC-3 is expressed in both isolectin-B4-binding (IB4) non-peptidergic, and peptidergic nociceptors (Bali et al., 2013; Pang et al., 2016). Although resting membrane potentials and voltage-dependent currents are unaltered after knockout or knockdown of CLC-3 and CLC-5, ablation of CLC-3 increases the excitability of DRG neurons as indicated by decreased AP thresholds and decreased rheobase (Pang et al., 2016; Qi et al., 2018). This is in line with the hypersensitivity to mechanical sensory stimulation in neuropathic and tumor pain models in rodents with a knockdown or genetic knockout of CLC-3 (Bali et al., 2013; Pang et al., 2016). Furthermore, CLC-3 and CLC-5 are involved in the downstream signaling of the biologically active sphingolipid S1P (Pang et al., 2016; Qi et al., 2018). S1P is released by platelets after surgery, trauma or blood vessel damage and induces nocifensive behavior in animal models *in vivo* and signatures of nociceptor activation in humans (Zhang et al., 2006; Mair et al., 2011; Camprubi-Robles et al., 2013; Li C. et al., 2015). As the cellular mechanisms underlying these S1P effects, we found that S1P directly depolarizes nociceptors through an excitatory inward current, which is significantly attenuated by siRNA-mediated CLC-3 or CLC-5 knockdown (Camprubi-Robles et al., 2013; Qi et al., 2018). The acute excitation of nociceptive neurons and the activation of CLC transporters involve S1P receptor type 3 (S1PR_3_)-dependent activation of the Rho GTPase signaling cascade, however, not Rho-associated protein kinase (ROCK) (Camprubi-Robles et al., 2013; Quarta et al., 2017; Qi et al., 2018; Kalpachidou et al., 2019). Importantly, these studies not only establish a role of CLC-3 and CLC-5 transporters in nociception, but whole-cell patch-clamp recordings also provide evidence that they affect conductances in the plasma membrane of neurons (Camprubi-Robles et al., 2013; Pang et al., 2016; Qi et al., 2018). Currently there is no data available whether CLC-3 and CLC-5 interact with accessory proteins and partially locate to the plasma membrane as found for certain splice variants in heterologous expression systems, or induce another Cl^–^ conductance (Kawasaki et al., 1995; Huang et al., 2001; Okada et al., 2014; Guzman et al., 2015; Jentsch and Pusch, 2018). After spared nerve injury, CLC-3 mRNA and protein levels surprisingly decrease in lumbar DRGs (Pang et al., 2016). In contrast to its acute excitatory action, the persisting suppression of CLC-3 expression correlates well with mechanical hypersensitivity which is rescued through intrathecal delivery of CLC-3 adenoviral vector (Pang et al., 2016). CLC-3 mRNA expression is also significantly reduced in DRGs in a cancer pain model, and siRNA knockdown of CLC-3 in DRGs further increases tumor-induced mechanical hyperalgesia (Bali et al., 2013).

#### CLC-6

CLC-6 is a 2Cl^–^/H^+^ exchanger in the membrane of (late) endosomes almost exclusively expressed in the nervous system (Poet et al., 2006). Its abundance is exceptionally high in trigeminal ganglia, DRGs and spinal cord suggesting a relevance of the transporter in the somatosensory system (Poet et al., 2006). Mice with a global depletion of CLC-6 exhibit dramatically increased tail-flick latencies in response to painful stimuli indicating severe deficits in nociception (Poet et al., 2006). As CLC-6 deficient mice do not show significant loss of neurons and only moderate other behavioral deficits, the nociceptive deficits most likely appear to be related to a disruption of neuronal functions through intracellular lysosomal deposition (Poet et al., 2006). However, the pathophysiological mechanisms causing the prominent impairment of nocifensive behavior caused by genetic deletion of CLC-6 are currently not understood.

### Cystic Fibrosis Transmembrane Conductance Regulator (CFTR)

Cystic fibrosis (CF) is a life-limiting autosomal recessive disorder caused by mutations in the gene encoding CFTR. CFTR is a member of the family of ATP binding cassette transporters, but is an anion channel that hydrolyzes ATP during the transport cycle of anions (e.g., Cl^–^, HCO_3_^–^), i.e., CFTR is an ATP-gated anion channel allowing for Cl^–^ flux along the electrochemical gradient (reviewed in Hwang and Kirk, 2013). CF-associated mutations mostly reduce CFTR channel function, giving rise to a multiplicity of symptoms in several organ systems and severe respiratory disease that is the major cause of death of CF patients. Although the main CF disease strains are non-neuronal, relevance of CFTR in neurons has been proposed for years, as the channel is expressed in the central and peripheral nervous system of several species, including rodents, pigs and humans (Mulberg et al., 1994, 1995, 1998; Rogan et al., 2010; Kanno and Nishizaki, 2011; Reznikov et al., 2013; Marcorelles et al., 2014; Reznikov, 2017). CFTR may be exclusively expressed in neurons but not glial cells in humans (Guo et al., 2009; Niu et al., 2009; Marcorelles et al., 2014). Importantly, evidence for CFTR-mediated anionic currents is not available for most cell types. However, there are some indications for functional expression of CFTR in DRG neurons and for its relevance in the development of mechanical allodynia (Kanno and Nishizaki, 2011). Indirect evidence suggests that noradrenalin may stimulate ATP release through CFTR after β3-adrenergic receptor stimulation as a mechanism to promote neuropathic pain (Kanno et al., 2010; Kanno and Nishizaki, 2011). Following activation of AMPA receptors in cultured spinal cord microglia, ATP release is strongly attenuated by pharmacological inhibition and genetic knockout of CFTR, indicating that CFTR may contribute to ATP release in spinal cord and probably also in DRGs (Liu et al., 2006; Kanno and Nishizaki, 2011). However, currently it is unknown whether these mechanisms are relevant for pain processing in nociceptors despite the undoubted significance of purinergic signaling in the entire pain pathway (e.g., Jahr and Jessell, 1983; reviewed in Burnstock, 2013). Functional, biophysical and pharmacological properties of CFTR in DRG and spinal cord neurons/glia remain elusive at present and pain phenotypes have not been conclusively reported in CF patients.

### Volume-Regulated Anion Channels (VRAC or VSOAC)

VRAC, also referred to as volume-sensitive organic osmolyte anion channel (VSOAC), mediates fluxes of anions and organic osmolytes (e.g., amino acids and their derivatives or methylamines). The channels accordingly control regulatory volume decrease (RVD) to compensate for cell swelling, changes of tonicity or intracellular ionic strength (for a comprehensive review see Jentsch et al., 2016). LRRC8 family members are the molecular correlates of VRAC, and LRRC8A constitutes an essential subunit which co-assembles with one or more other LRRC8 member(s) to give rise to VRAC (Qiu et al., 2014; Voss et al., 2014). DRG neurons express mRNA transcripts encoding LRRC8A and a VRAC current can be induced both by hypotonic extracellular solution as well as by hypertonic pipette solution suggesting that LRRC8A contributes to VRAC in DRG neurons, albeit a role in nociception has not been shown unequivocally (Wang et al., 2017; Liu et al., 2019). Interestingly, Ca^2+^-activated Cl^–^ channels might contribute to VRAC: Ano1 can also be activated by cell swelling and members of the tweety-homolog family (see Ca^2+^-activated Cl^–^ channel section) constitute essential subunits for swelling-induced VRAC in cultured cortical astrocytes (Han et al., 2019; Liu et al., 2019). As mRNA transcripts encoding Ttyh1/2/3 are apparently abundant in lumbar DRGs, and as expression levels of Ttyh1 appear to be slightly regulated in axotomized DRG neurons, it is tempting to speculate that tweety proteins may contribute to VRAC currents in DRG neurons (L4/L5; Al-Jumaily et al., 2007; Boudes et al., 2009). However, functional expression of these proteins has not been explored in the somatosensory system yet.

### Maxi-Anion Channels (Also Maxi-Cl^–^ Channels)

Maxi-anion channels (MACs) exhibit extraordinarily large unitary conductances (200–500 pS), and probably are ubiquitously expressed in virtually every cell type (reviewed in Sabirov et al., 2016). Apart from providing transfer routes for anions, MACs mediate release pathways for ATP and are associated with purinergic signaling (e.g., Bell et al., 2003; Sabirov and Okada, 2005). In resting cells, MACs are usually inactive, but can be activated by a multitude of stimuli including cell stress (osmotic, ionic strength, mechanical, heat, oxidation, etc.). Further, MAC activity is modulated in context of GPCR signaling through several ligands such as endothelin-1, adenosine, and bradykinin, and inhibited by PGE_2_ (summarized in Sabirov et al., 2016, 2017). The prostaglandin transporter SLCO2A1 has been identified as the molecular correlate of MACs (Kanai et al., 1995; Sabirov et al., 2017). As SLCO2A1/MAC activity is modulated by a large number of pain-initiating and pro-inflammatory stimuli (e.g., cell damage, ligands of GPCRs, prostaglandins, etc.) and given its involvement in purinergic signaling, these channels may be of relevance in peripheral pain mechanisms. However, the abundance and function of SLCO2A1 in the peripheral nervous system has not been addressed yet.

## Clinical Potential of Chloride channels/transporters for Pain Therapy

As nociceptors are the primary sensors for noxious stimuli and the site of peripheral sensitization before subsequent changes in the entire pain pathway manifest chronification, they remain an attractive target site for pain therapies. Multiple studies demonstrate that painful conditions lead to changes in various Cl^–^ channels, transporters and homeostasis in primary afferent nociceptors, while *vice versa* altered activity of Cl^–^ channels and transporters affects pain perception. This draws attention to nociceptor chloride channels and transporters as potential target for the development of analgesic drugs. Bumetanide, a loop diuretic which targets the kidney-specific NKCC2, also prevents the accumulation of phosphorylated NKCC1 in DRGs, as well as the concomitant downregulation of KCC2 in the spinal cord, and alleviates mechanical and thermal hypersensitivity in a model for neuropathic pain (Modol et al., 2014). Similarly, compensating the CCC changes by viral delivery of KCC2 into DRG and spinal neurons reverses the depolarizing shift of the reversal potential for GABA-mediated currents and abolishes hypersensitivity induced by nerve injury (Li et al., 2016). Although these studies indicate great potential for targeting Cl^–^ homeostasis in pain conditions, they have to be considered carefully since Cl^–^ homeostasis mechanisms and nociceptor function may not be fully identical in rodents and humans (Funk et al., 2008; Rocha-Gonzalez et al., 2008; Zhang et al., 2018).

Besides, Cl^–^ channels expressed in primary afferent nociceptors can be targeted for potential pain treatment. However, this is complicated first by the previously described variability of the Cl^–^ reversal potential, which can switch the effect of Cl^–^ channel opening from inhibitory to excitatory and thus profound control of Cl^–^ concentration is indispensable, and second by the involvement of Cl^–^ conductances in other physiological functions. Notably, Best1 and Ano1 play an important role in retina and secretory epithelia function, respectively (Hartzell et al., 2008; Oh and Jung, 2016). To overcome the problems of potential unwanted effects, splice variant- or subunit-specific drug development may offer promising perspectives: α1-sparing GABA_A_ receptor agonists alleviate hyperalgesia induced by inflammation or chronic nerve constriction through pre- and postsynaptic action at nociceptive synapses in the SDH without a sedating effect (Knabl et al., 2008; Di Lio et al., 2011; Witschi et al., 2011). Likewise, α3 subunits of GlyRs moved into the focus of analgesic drug discovery, since they are targeted by cannabinoids and mediate analgesic properties. Although their primary action seems to be at the SDH, α3 GlyRs are also expressed in DRG neurons, and these might contribute to the analgesic effect (Lynch et al., 2017). Thus, Cl^–^ channels on primary afferents can be targeted to achieve analgesia also in chronic pain conditions, but warrant further developments for pain relief in humans.

## Synopsis

Cl^–^ homeostasis is tightly regulated in nociceptive primary afferents and essential for the effect of activation of Cl^–^ channels on the excitability of these neurons. In contrast to the ‘static’ action of cation flux on cellular membrane potential and excitability, the Cl^–^ reversal potential in primary afferent neurons typically lies between the resting membrane potential and the AP threshold and is subject to fluctuation during persisting pain. This can convert the normally inhibitory action of Cl^–^ channel opening into an excitatory one contributing to pain, allodynia, and hyperalgesia. Not only Cl^–^ concentrations but also Cl^–^ channels and transporters expressed in DRG neurons are deregulated in pain disorders (see Figure 1). A distinct role and mode of activation in nociceptors is already established for some of them, like GABA_A_ receptors or Ano1, while the importance of others such as tweety homologs or CICA has not been explored although they are detectable in DRGs. Altogether, Cl^–^ ions are emerging as relevant components of nociceptor function and should no longer be underrated as important players in acute pain processing and the pathogenesis of chronic pain disorders.

## Author Contributions

All authors listed have made a substantial contribution to writing the manuscript, revised it and approved it for publication. BW designed the figures.

## Conflict of Interest

The authors declare that the research was conducted in the absence of any commercial or financial relationships that could be construed as a potential conflict of interest.

## Abbreviations

5-HT: serotonin
Ano: anoctamin
AP: action potential
Best: bestrophin
CCC: cation-chloride cotransporter
CF: cystic fibrosis
CFTR: cystic fibrosis transmembrane conductance regulator
ClCA: chloride channel accessory
IP_3_: Inositol-1,4,5-trisphosphate
SDH: dorsal horn of the spinal cord
GABA: γ -aminobutyric acid
GlyR: glycine receptor
GPCR: G-protein coupled receptor
KCC: K^+^-Cl^–^ Cotransporter
MAC: maxi-anion channel
NKCC: Na^+^-K^+^-2Cl^–^ cotransporter
PGE_2_: prostaglandin E2
PIP_2_: phosphatidylinositol-4,5-bisphosphate
S1P: sphingosine-1-phosphate
VGCC: voltage-gated calcium channels
VRAC: volume-regulated anion channel
VSOAC: volume-sensitive organic osmolyte anion channel.

## References

[B1] Ahmadi S. Lippross S. Neuhuber W. L. Zeilhofer H. U. (2002). PGE(2) selectively blocks inhibitory glycinergic neurotransmission onto rat superficial dorsal horn neurons. *Nat. Neurosci.* 5 34–40. 10.1038/nn778 11740501

[B2] Akasu T. Yamada K. (1997). Neurokinin-1 (NK1) receptors mediate tachykinin-induced depression of GABA current in bullfrog sensory neurons. *Kurume Med. J.* 44 33–41. 10.2739/kurumemedj.44.33 9154760

[B3] Al-Jumaily M. Kozlenkov A. Mechaly I. Fichard A. Matha V. Scamps F. (2007). Expression of three distinct families of calcium-activated chloride channel genes in the mouse dorsal root ganglion. *Neurosci. Bull.* 23 293–299. 10.1007/s12264-007-0044-8 17952139 PMC5550578

[B4] André S. Boukhaddaoui H. Campo B. Al-Jumaily M. Mayeux V. Greuet D. (2003). Axotomy-induced expression of calcium-activated chloride current in subpopulations of mouse dorsal root ganglion neurons. *J. Neurophysiol.* 90 3764–3773. 10.1152/jn.00449.2003 12944538

[B5] Bali K. K. Selvaraj D. Satagopam V. P. Lu J. Schneider R. Kuner R. (2013). Genome-wide identification and functional analyses of microRNA signatures associated with cancer pain. *EMBO Mol. Med.* 5 1740–1758. 10.1002/emmm.201302797 24039159 PMC3840489

[B6] Barragan-Iglesias P. Rocha-Gonzalez H. I. Pineda-Farias J. B. Murbartian J. Godinez-Chaparro B. Reinach P. S. (2014). Inhibition of peripheral anion exchanger 3 decreases formalin-induced pain. *Eur. J. Pharmacol.* 738 91–100. 10.1016/j.ejphar.2014.05.029 24877687

[B7] Basbaum A. I. Bautista D. M. Scherrer G. Julius D. (2009). Cellular and molecular mechanisms of pain. *Cell* 139 267–284. 10.1016/j.cell.2009.09.028 19837031 PMC2852643

[B8] Bell P. D. Lapointe J. Y. Sabirov R. Hayashi S. Peti-Peterdi J. Manabe K. (2003). Macula densa cell signaling involves ATP release through a maxi anion channel. *Proc. Natl. Acad. Sci. U.S.A.* 100 4322–4327. 10.1073/pnas.0736323100 12655045 PMC153091

[B9] Ben-Ari Y. Khalilov I. Kahle K. T. Cherubini E. (2012). The GABA excitatory/inhibitory shift in brain maturation and neurological disorders. *Neuroscientist* 18 467–486. 10.1177/1073858412438697 22547529

[B10] Betelli C. MacDermott A. B. Bardoni R. (2015). Transient, activity dependent inhibition of transmitter release from low threshold afferents mediated by GABAA receptors in spinal cord lamina III/IV. *Mol. Pain* 11:64. 10.1186/s12990-015-0067-5 26463733 PMC4605127

[B11] Bharill S. Fu Z. Palty R. Isacoff E. Y. (2014). Stoichiometry and specific assembly of Best ion channels. *Proc. Natl. Acad. Sci. U.S.A.* 111 6491–6496. 10.1073/pnas.1400248111 24748110 PMC4035945

[B12] Bhat R. Axtell R. Mitra A. Miranda M. Lock C. Tsien R. W. (2010). Inhibitory role for GABA in autoimmune inflammation. *Proc. Natl. Acad. Sci. U.S.A.* 107 2580–2585. 10.1073/pnas.0915139107 20133656 PMC2823917

[B13] Bhisitkul R. B. Kocsis J. D. Gordon T. R. Waxman S. G. (1990). Trophic influence of the distal nerve segment on GABAA receptor expression in axotomized adult sensory neurons. *Exp. Neurol.* 109 273–278. 10.1016/s0014-4886(05)80017-2 2170161

[B14] Bhisitkul R. B. Villa J. E. Kocsis J. D. (1987). Axonal GABA receptors are selectively present on normal and regenerated sensory fibers in rat peripheral nerve. *Exp. Brain Res.* 66 659–663. 10.1007/bf00270698 3038587

[B15] Bormann J. (2000). The ABC of GABA receptors. *Trends Pharmacol. Sci.* 21 16–19. 10.1016/s0165-6147(99)01413-3 10637650

[B16] Boudes M. Sar C. Menigoz A. Hilaire C. Pequignot M. O. Kozlenkov A. (2009). Best1 is a gene regulated by nerve injury and required for Ca2+-activated Cl- current expression in axotomized sensory neurons. *J. Neurosci.* 29 10063–10071. 10.1523/JNEUROSCI.1312-09.2009 19675239 PMC2761749

[B17] Bourinet E. Zamponi G. W. (2005). Voltage gated calcium channels as targets for analgesics. *Curr. Top. Med. Chem* 5 539–546. 10.2174/1568026054367610 16022676

[B18] Bravo-Hernandez M. Corleto J. A. Barragan-Iglesias P. Gonzalez-Ramirez R. Pineda-Farias J. B. Felix R. (2016). The alpha5 subunit containing GABAA receptors contribute to chronic pain. *Pain* 157 613–626. 10.1097/j.pain.0000000000000410 26545088 PMC4950669

[B19] Bravo-Hernandez M. Feria-Morales L. A. Torres-Lopez J. E. Cervantes-Duran C. Delgado-Lezama R. Granados-Soto V. (2014). Evidence for the participation of peripheral alpha5 subunit-containing GABAA receptors in GABAA agonists-induced nociception in rats. *Eur. J. Pharmacol.* 734 91–97. 10.1016/j.ejphar.2014.03.051 24726872

[B20] Budai D. Fields H. L. (1998). Endogenous opioid peptides acting at mu-opioid receptors in the dorsal horn contribute to midbrain modulation of spinal nociceptive neurons. *J. Neurophysiol.* 79 677–687. 10.1152/jn.1998.79.2.677 9463431

[B21] Burnstock G. (2013). Purinergic mechanisms and pain–an update. *Eur. J. Pharmacol.* 716 24–40. 10.1016/j.ejphar.2013.01.078 23524093

[B22] Camprubi-Robles M. Mair N. Andratsch M. Benetti C. Beroukas D. Rukwied R. (2013). Sphingosine-1-phosphate-induced nociceptor excitation and ongoing pain behavior in mice and humans is largely mediated by S1P3 receptor. *J. Neurosci.* 33 2582–2592. 10.1523/JNEUROSCI.4479-12.2013 23392686 PMC6619173

[B23] Caputo A. Caci E. Ferrera L. Pedemonte N. Barsanti C. Sondo E. (2008). TMEM16A, a membrane protein associated with calcium-dependent chloride channel activity. *Science* 322 590–594. 10.1126/science.1163518 18772398

[B24] Carlton S. M. Zhou S. Coggeshall R. E. (1999). Peripheral GABA(A) receptors: evidence for peripheral primary afferent depolarization. *Neuroscience* 93 713–722. 10.1016/s0306-4522(99)00101-3 10465455

[B25] Chakrabarti S. Pattison L. A. Singhal K. Hockley J. R. F. Callejo G. Smith E. S. J. (2018). Acute inflammation sensitizes knee-innervating sensory neurons and decreases mouse digging behavior in a TRPV1-dependent manner. *Neuropharmacology* 143 49–62. 10.1016/j.neuropharm.2018.09.014 30240782 PMC6277850

[B26] Chen J. T. Guo D. Campanelli D. Frattini F. Mayer F. Zhou L. (2014). Presynaptic GABAergic inhibition regulated by BDNF contributes to neuropathic pain induction. *Nat. Commun.* 5:5331. 10.1038/ncomms6331 25354791 PMC4220496

[B27] Chen Q. Y. Tan C. Y. Wang Y. Ma K. T. Li L. Si J. Q. (2019). Mechanism of persistent hyperalgesia in neuropathic pain caused by chronic constriction injury. *Neural Regen. Res.* 14 1091–1098. 10.4103/1673-5374.250631 30762024 PMC6404508

[B28] Cho H. Yang Y. D. Lee J. Lee B. Kim T. Jang Y. (2012). The calcium-activated chloride channel anoctamin 1 acts as a heat sensor in nociceptive neurons. *Nat. Neurosci.* 15 1015–1021. 10.1038/nn.3111 22634729

[B29] Cordero-Erausquin M. Coull J. A. M. Boudreau D. Rolland M. De Koninck Y. (2005). Differential maturation of GABA action and anion reversal potential in spinal lamina I neurons: impact of chloride extrusion capacity. *J. Neurosci.* 25 9613–9623. 10.1523/jneurosci.1488-05.2005 16237166 PMC6725724

[B30] Coull J. A. M. Boudreau D. Bachand K. Prescott S. A. Nault F. Sík A. (2003). Trans-synaptic shift in anion gradient in spinal lamina I neurons as a mechanism of neuropathic pain. *Nature* 424 938–942. 10.1038/nature01868 12931188

[B31] De Groat W. C. Lalley P. M. Saum W. R. (1972). Depolarization of dorsal root ganglia in the cat by GABA and related amino acids: antagonism by picrotoxin and bicuculline. *Brain Res.* 44 273–277. 10.1016/0006-8993(72)90383-6 4341462

[B32] De la Luz-Cuellar Y. E. Rodriguez-Palma E. J. Franco-Enzastiga U. Salinas-Abarca A. B. Delgado-Lezama R. Granados-Soto V. (2019). Blockade of spinal alpha5-GABAA receptors differentially reduces reserpine-induced fibromyalgia-type pain in female rats. *Eur. J. Pharmacol.* 858:172443. 10.1016/j.ejphar.2019.172443 31181208

[B33] Deba F. Bessac B. F. (2015). Anoctamin-1 Cl- channels in nociception: activation by an N-aroylaminothiazole and capsaicin and inhibition by T16A[inh]-A01. *Mol. Pain* 11:12990. 10.1186/s12990-015-0061-y 26364309 PMC4567824

[B34] Desarmenien M. Feltz P. Headley P. M. (1979). The depolarizing responses to GABA in rat sensory ganglia in vivo and in vitro. A study of the role of glial uptake. *J. Physiol.* 75 661–665. 232721

[B35] Desarmenien M. Feltz P. Headley P. M. (1980). Does glial uptake affect GABA responses? AN intracellular study on rat dorsal root ganglion neurones in vitro. *J. Physiol.* 307 163–182. 10.1113/jphysiol.1980.sp013429 6259333 PMC1283039

[B36] Desarmenien M. Santangelo F. Linck G. Headley P. M. Feltz P. (1981). Physiological study of amino acid uptake and receptor desensitization: the GABA system in dorsal root ganglia. *Adv. Biochem. Psychopharmacol.* 29 309–319. 6266216

[B37] Di Lio A. Benke D. Besson M. Desmeules J. Daali Y. Wang Z. J. (2011). HZ166, a novel GABAA receptor subtype-selective benzodiazepine site ligand, is antihyperalgesic in mouse models of inflammatory and neuropathic pain. *Neuropharmacology* 60 626–632. 10.1016/j.neuropharm.2010.11.026 21145329 PMC3566476

[B38] Ding J. Delpire E. (2014). Deletion of KCC3 in parvalbumin neurons leads to locomotor deficit in a conditional mouse model of peripheral neuropathy associated with agenesis of the corpus callosum. *Behav. Brain Res.* 274 128–136. 10.1016/j.bbr.2014.08.005 25116249 PMC4179972

[B39] Doyon N. Prescott S. A. Castonguay A. Godin A. G. Kröger H. De Koninck Y. (2011). Efficacy of synaptic inhibition depends on multiple, dynamically interacting mechanisms implicated in chloride homeostasis. *PLoS Comput. Biol.* 7:e1002149. 10.1371/journal.pcbi.1002149 21931544 PMC3169517

[B40] Doyon N. Vinay L. Prescott S. A. Koninck Y. D. De Koninck Y. (2016). Chloride regulation: a dynamic equilibrium crucial for synaptic inhibition. *Neuron* 89 1157–1172. 10.1016/j.neuron.2016.02.030 26985723

[B41] Du X. Hao H. Yang Y. Huang S. Wang C. Gigout S. (2017). Local GABAergic signaling within sensory ganglia controls peripheral nociceptive transmission. *J. Clin. Invest.* 127 1741–1756. 10.1172/JCI86812 28375159 PMC5409786

[B42] Dutertre S. Becker C. M. Betz H. (2012). Inhibitory glycine receptors: an update. *J. Biol. Chem.* 287 40216–40223. 10.1074/jbc.R112.408229 23038260 PMC3504737

[B43] Ebbinghaus M. Gajda M. Holtzman M. J. Schulz S. Schaible H.-G. (2014). Does chloride channel accessory 3 have a role in arthritis pain? A study on murine antigen-induced arthritis. *Neurosci. Lett.* 576 40–44. 10.1016/j.neulet.2014.05.051 24905173

[B44] Enna S. J. McCarson K. E. (2006). The role of GABA in the mediation and perception of pain. *Adv. Pharmacol.* 54 1–27. 10.1016/s1054-3589(06)54001-3 17175808

[B45] Ertongur-Fauth T. Hochheimer A. Buescher J. M. Rapprich S. Krohn M. (2014). A novelTMEM16Asplice variant lacking the dimerization domain contributes to calcium-activated chloride secretion in human sweat gland epithelial cells. *Exp. Dermatol.* 23 825–831. 10.1111/exd.12543 25220078

[B46] Evans S. R. Thoreson W. B. Beck C. L. (2004). Molecular and functional analyses of two new calcium-activated chloride channel family members from mouse eye and intestine. *J. Biol. Chem.* 279 41792–41800. 10.1074/jbc.m408354200 15284223 PMC1383427

[B47] Ferrera L. Caputo A. Ubby I. Bussani E. Zegarra-Moran O. Ravazzolo R. (2009). Regulation of TMEM16A chloride channel properties by alternative splicing. *J. Biol. Chem.* 284 33360–33368. 10.1074/jbc.M109.046607 19819874 PMC2785179

[B48] Fischmeister R. Hartzell H. C. (2005). Volume sensitivity of the bestrophin family of chloride channels. *J. Physiol.* 562 477–491. 10.1113/jphysiol.2004.075622 15564283 PMC1665509

[B49] Flegel C. Schöbel N. Altmüller J. Becker C. Tannapfel A. Hatt H. (2015). RNA-Seq analysis of human trigeminal and dorsal root ganglia with a focus on chemoreceptors. *PLoS One* 10:e0128951. 10.1371/journal.pone.0128951 26070209 PMC4466559

[B50] Francois A. Low S. A. Sypek E. I. Christensen A. J. Sotoudeh C. Beier K. T. (2017). A brainstem-spinal cord inhibitory circuit for mechanical pain modulation by GABA and enkephalins. *Neuron* 93:82. 10.1016/j.neuron.2017.01.008 28162807 PMC7354674

[B51] Funk K. Woitecki A. Franjic-Würtz C. Gensch T. Möhrlen F. Frings S. (2008). Modulation of chloride homeostasis by inflammatory mediators in dorsal root ganglion neurons. *Mol. Pain* 4 1–12. 10.1186/1744-8069-4-32 18700020 PMC2526990

[B52] Furuyama T. Sato M. Sato K. Araki T. Inagaki S. Takagi H. (1992). Co-expression of glycine receptor beta subunit and GABAA receptor gamma subunit mRNA in the rat dorsal root ganglion cells. *Brain Res. Mol. Brain Res.* 12 335–338. 10.1016/0169-328x(92)90136-y 1315905

[B53] Galaz P. Barra R. Figueroa H. Mariqueo T. (2015). Advances in the pharmacology of lGICs auxiliary subunits. *Pharmacol. Res.* 101 65–73. 10.1016/j.phrs.2015.07.026 26255765

[B54] Gallagher J. P. Nakamura J. Shinnick-Gallagher P. (1983a). Effects of glial uptake and desensitization on the activity of gamma-aminobutyric acid (GABA) and its analogs at the cat dorsal root ganglion. *J. Pharmacol. Exp. Ther.* 226 876–884. 6310083

[B55] Gallagher J. P. Nakamura J. Shinnick-Gallagher P. (1983b). The effects of temperature, pH and Cl-pump inhibitors on GABA responses recorded from cat dorsal root ganglia. *Brain Res.* 267 249–259. 10.1016/0006-8993(83)90877-6 6307467

[B56] Gamba G. (2005). Molecular physiology and pathophysiology of electroneutral cation-chloride cotransporters. *Physiol. Rev.* 85 423–493. 10.1152/physrev.00011.2004 15788703

[B57] Gandhi R. Elble R. C. Gruber A. D. Schreur K. D. Ji H. L. Fuller C. M. (1998). Molecular and functional characterization of a calcium-sensitive chloride channel from mouse lung. *J. Biol. Chem.* 273 32096–32101. 10.1074/jbc.273.48.32096 9822685

[B58] García G. Martínez-Rojas V. A. Rocha-González H. I. Granados-Soto V. Murbartián J. (2014). Evidence for the participation of Ca2+-activated chloride channels in formalin-induced acute and chronic nociception. *Brain Res.* 1579 35–44. 10.1016/j.brainres.2014.07.011 25036442

[B59] Gibson A. Lewis A. P. Affleck K. Aitken A. J. Meldrum E. Thompson N. (2005). hCLCA1 and mCLCA3 are secreted non-integral membrane proteins and therefore are not ion channels. *J. Biol. Chem.* 280 27205–27212. 10.1074/jbc.m504654200 15919655

[B60] Gilbert D. Franjic-Würtz C. Funk K. Gensch T. Frings S. Möhrlen F. (2007). Differential maturation of chloride homeostasis in primary afferent neurons of the somatosensory system. *Intern. J. Dev. Neurosci.* 25 479–489. 10.1016/j.ijdevneu.2007.08.001 17869474

[B61] Golubinskaya V. Vontell R. Supramaniam V. Gustafsson H. Mallard C. Nilsson H. (2019). Bestrophin-3 expression in a subpopulation of astrocytes in the neonatal brain after hypoxic-ischemic injury. *Front. Physiol.* 10:23. 10.3389/fphys.2019.00023 30761013 PMC6362097

[B62] Grubb S. Poulsen K. A. Juul C. A. Kyed T. Klausen T. K. Larsen E. H. (2013). TMEM16F (Anoctamin 6), an anion channel of delayed Ca2+activation. *J. Gen. Physiol.* 141 585–600. 10.1085/jgp.201210861 23630341 PMC3639583

[B63] Gruber A. D. Elble R. C. Ji H.-L. Schreur K. D. Fuller C. M. Pauli B. U. (1998). Genomic cloning, molecular characterization, and functional analysis of human clca1, the first human member of the family of Ca2+-activated Cl-channel proteins. *Genomics* 54 200–214. 10.1006/geno.1998.5562 9828122

[B64] Gruber A. D. Schreur K. D. Ji H.-L. Fuller C. M. Pauli B. U. (1999). Molecular cloning and transmembrane structure of hCLCA2 from human lung, trachea, and mammary gland. *Am. J. Physiol. Cell Physiol.* 276 C1261–C1270. 10.1152/ajpcell.1999.276.6.C1261 10362588

[B65] Guan B. C. Li Z. W. Zhou X. P. (1994). Modulatory effects of substance P on the membrane responses mediated by GABAA and GABAB receptors in DRG neurons. *Sheng Li Xue Bao* 46 441–450. 7531369

[B66] Gulledge A. T. Stuart G. J. (2003). Excitatory actions of GABA in the cortex. *Neuron* 37 299–309. 10.1016/s0896-6273(02)01146-7 12546824

[B67] Guo Y. Su M. Su M. McNutt M. A. Gu J. (2009). Expression and distribution of cystic fibrosis transmembrane conductance regulator in neurons of the spinal cord. *J. Neurosci. Res.* 87 3611–3619. 10.1002/jnr.22154 19533735 PMC7167064

[B68] Guzman R. E. Miranda-Laferte E. Franzen A. Fahlke C. (2015). Neuronal ClC-3 splice variants differ in subcellular localizations, but mediate identical transport functions. *J. Biol. Chem.* 290 25851–25862. 10.1074/jbc.M115.668186 26342074 PMC4646242

[B69] Han Y.-E. Kwon J. Won J. An H. Jang M. W. Woo J. (2019). Tweety-homolog (Ttyh) family encodes the pore-forming subunits of the swelling-dependent volume-regulated anion channel (VRACswell) in the Brain. *Exp. Neurobiol.* 28:183. 10.5607/en.2019.28.2.183 31138989 PMC6526117

[B70] Hartzell H. C. Qu Z. Yu K. Xiao Q. Chien L.-T. (2008). Molecular physiology of bestrophins: multifunctional membrane proteins linked to best disease and other retinopathies. *Physiol. Rev.* 88 639–672. 10.1152/physrev.00022.2007 18391176

[B71] Harvey R. J. Depner U. B. Wassle H. Ahmadi S. Heindl C. Reinold H. (2004). GlyR alpha3: an essential target for spinal PGE2-mediated inflammatory pain sensitization. *Science* 304 884–887. 10.1126/science.1094925 15131310

[B72] Heinricher M. M. Tavares I. Leith J. L. Lumb B. M. (2009). Descending control of nociception: Specificity, recruitment and plasticity. *Brain Res. Rev.* 60 214–225. 10.1016/j.brainresrev.2008.12.009 19146877 PMC2894733

[B73] Hu H. Z. Li Z. W. (1997). Modulation by adenosine of GABA-activated current in rat dorsal root ganglion neurons. *J. Physiol.* 501(Pt 1), 67–75. 10.1111/j.1469-7793.1997.067bo.x 9174995 PMC1159505

[B74] Huang P. Liu J. Di A. Robinson N. C. Musch M. W. Kaetzel M. A. (2001). Regulation of human CLC-3 channels by multifunctional Ca2+/calmodulin-dependent protein kinase. *J. Biol. Chem.* 276 20093–20100. 10.1074/jbc.m009376200 11274166

[B75] Hwang T. C. Kirk K. L. (2013). The CFTR ion channel: gating, regulation, and anion permeation. *Cold Spring Harb. Perspect. Med.* 3:a009498. 10.1101/cshperspect.a009498 23284076 PMC3530039

[B76] Imhof A.-K. Glück L. Gajda M. Bräuer R. Schaible H.-G. Schulz S. (2011). Potent anti-inflammatory and antinociceptive activity of the endothelin receptor antagonist bosentan in monoarthritic mice. *Arthrit. Res. Ther.* 13:R97. 10.1186/ar3372 21689431 PMC3218912

[B77] Jahr C. E. Jessell T. M. (1983). ATP excites a subpopulation of rat dorsal horn neurones. *Nature* 304 730–733. 10.1038/304730a0 6888539

[B78] Jang I. J. Davies A. J. Akimoto N. Back S. K. Lee P. R. Na H. S. (2017). Acute inflammation reveals GABAA receptor-mediated nociception in mouse dorsal root ganglion neurons via PGE2 receptor 4 signaling. *Physiol. Rep.* 5:e13178. 10.14814/phy2.13178 28438981 PMC5408276

[B79] Jennings E. M. Okine B. N. Roche M. Finn D. P. (2014). Stress-induced hyperalgesia. *Prog. Neurobiol.* 121 1–18. 10.1016/j.pneurobio.2014.06.003 25010858

[B80] Jentsch T. J. Lutter D. Planells-Cases R. Ullrich F. Voss F. K. (2016). VRAC: molecular identification as LRRC8 heteromers with differential functions. *Pflugers. Arch.* 468 385–393. 10.1007/s00424-015-1766-5 26635246

[B81] Jentsch T. J. Poet M. Fuhrmann J. C. Zdebik A. A. (2005). Physiological functions of CLC Cl- channels gleaned from human genetic disease and mouse models. *Annu. Rev. Physiol.* 67 779–807. 10.1146/annurev.physiol.67.032003.153245 15709978

[B82] Jentsch T. J. Pusch M. (2018). CLC chloride channels and transporters: structure, function, physiology, and disease. *Physiol. Rev.* 98 1493–1590. 10.1152/physrev.00047.2017 29845874

[B83] Jentsch T. J. Steinmeyer K. Schwarz G. (1990). Primary structure of torpedo marmorata chloride channel isolated by expression cloning in *Xenopus oocytes*. *Nature* 348 510–514. 10.1038/348510a0 2174129

[B84] Jin X. Shah S. Du X. Zhang H. Gamper N. (2016). Activation of Ca(2+) -activated Cl(-) channel ANO1 by localized Ca(2+) signals. *J. Physiol.* 594 19–30. 10.1113/jphysiol.2014.275107 25398532 PMC4704509

[B85] Jin X. Shah S. Liu Y. Zhang H. Lees M. Fu Z. (2013). Activation of the Cl- channel ANO1 by localized calcium signals in nociceptive sensory neurons requires coupling with the IP3 receptor. *Sci. Signal.* 6:ra73. 10.1126/scisignal.2004184 23982204 PMC4135425

[B86] Kaila K. Price T. J. Payne J. A. Puskarjov M. Voipio J. (2014). Cation-chloride cotransporters in neuronal development, plasticity and disease. *Nat. Rev. Neurosci.* 15 637–654. 10.1038/nrn3819 25234263 PMC4294553

[B87] Kalpachidou T. Spiecker L. Kress M. Quarta S. (2019). Rho GTPases in the physiology and pathophysiology of peripheral sensory neurons. *Cells* 8:591. 10.3390/cells8060591 31208035 PMC6627758

[B88] Kanai N. Lu R. Satriano J. A. Bao Y. Wolkoff A. W. Schuster V. L. (1995). Identification and characterization of a prostaglandin transporter. *Science* 268 866–869. 10.1126/science.7754369 7754369

[B89] Kanaka C. Ohno K. Okabe A. Kuriyama K. Itoh T. Fukuda A. (2001). The differential expression patterns of messenger RNAs encoding K-Cl cotransporters (KCC1,2) and Na-K-2Cl cotransporter (NKCC1) in the rat nervous system. *Neuroscience* 104 933–946. 10.1016/s0306-4522(01)00149-x 11457581

[B90] Kane Dickson V. Pedi L. Long S. B. (2014). Structure and insights into the function of a Ca2+-activated Cl- channel. *Nature* 516 213–218. 10.1038/nature13913 25337878 PMC4454446

[B91] Kanno T. Nishizaki T. (2011). CFTR mediates noradrenaline-induced ATP efflux from DRG neurons. *Mol. Pain* 7:72. 10.1186/1744-8069-7-72 21943397 PMC3192679

[B92] Kanno T. Yaguchi T. Nishizaki T. (2010). Noradrenaline stimulates ATP release from DRG neurons by targeting beta(3) adrenoceptors as a factor of neuropathic pain. *J. Cell. Physiol.* 224 345–351. 10.1002/jcp.22114 20432431

[B93] Kawasaki M. Suzuki M. Uchida S. Sasaki S. Marumo F. (1995). Stable and functional expression of the CIC-3 chloride channel in somatic cell lines. *Neuron* 14 1285–1291. 10.1016/0896-6273(95)90275-9 7605637

[B94] Knabl J. Witschi R. Hosl K. Reinold H. Zeilhofer U. B. Ahmadi S. (2008). Reversal of pathological pain through specific spinal GABAA receptor subtypes. *Nature* 451 330–334. 10.1038/nature06493 18202657

[B95] Kullmann D. M. Ruiz A. Rusakov D. M. Scott R. Semyanov A. Walker M. C. (2005). Presynaptic, extrasynaptic and axonal GABAA receptors in the CNS: where and why? *Prog. Biophys. Mol. Biol.* 87 33–46. 10.1016/j.pbiomolbio.2004.06.003 15471589 PMC3369532

[B96] Kuner T. Augustine G. J. (2000). A genetically encoded ratiometric indicator for chloride: capturing chloride transients in cultured hippocampal neurons. *Neuron* 27 447–459. 10.1016/s0896-6273(00)00056-8 11055428

[B97] Kuo Y.-H. Abdullaev I. F. Hyzinski-García M. C. Mongin A. A. (2014). Effects of alternative splicing on the function of bestrophin-1 calcium-activated chloride channels. *Biochem. J.* 458 575–583. 10.1042/bj20121546 24341532 PMC4145631

[B98] Labrakakis C. Tong C. K. Weissman T. Torsney C. MacDermott A. B. (2003). Localization and function of ATP and GABAA receptors expressed by nociceptors and other postnatal sensory neurons in rat. *J. Physiol.* 549(Pt 1), 131–142. 10.1113/jphysiol.2002.031963 12665615 PMC2342927

[B99] Le S. C. Jia Z. Chen J. Yang H. (2019). Molecular basis of PIP2-dependent regulation of the Ca2+-activated chloride channel TMEM16A. *Nat. Commun.* 10:3769. 10.1038/s41467-019-11784-8 31434906 PMC6704070

[B100] Lee B. Cho H. Jung J. Yang Y. D. Yang D.-J. Oh U. (2014). Anoctamin 1 contributes to inflammatory and nerve-injury induced hypersensitivity. *Mol. Pain* 10 1710–1745. 10.1186/1744-8069-10-5 PMC3929161 24450308

[B101] Lee K. Y. Charbonnet M. Gold M. S. (2012). Upregulation of high-affinity GABA(A) receptors in cultured rat dorsal root ganglion neurons. *Neuroscience* 208 133–142. 10.1016/j.neuroscience.2012.01.050 22366297 PMC3311786

[B102] Lee S. Yoon B. E. Berglund K. Oh S. J. Park H. Shin H. S. (2010). Channel-mediated tonic GABA release from Glia. *Science* 330 790–796. 10.1126/science.1184334 20929730

[B103] Li C. Li J. N. Kays J. Guerrero M. Nicol G. D. (2015). Sphingosine 1-phosphate enhances the excitability of rat sensory neurons through activation of sphingosine 1-phosphate receptors 1 and/or 3. *J. Neuroinflamm.* 12:70. 10.1186/s12974-015-0286-8 PMC4397880 25880547

[B104] Li L. Zhao L. Wang Y. Ma K. T. Shi W. Y. Wang Y. Z. (2015). PKCvarepsilon mediates substance P inhibition of GABAA receptors-mediated current in rat dorsal root ganglion. *J. Huazhong Univ. Sci. Technolog. Med. Sci.* 35 1–9. 10.1007/s11596-015-1380-y 25673185

[B105] Li J. Blankenship M. L. Baccei M. L. (2013). Deficits in glycinergic inhibition within adult spinal nociceptive circuits after neonatal tissue damage. *Pain* 154 1129–1139. 10.1016/j.pain.2013.03.030 23639821 PMC3795070

[B106] Li L. Chen S. R. Chen H. Wen L. Hittelman W. N. Xie J. D. (2016). Chloride homeostasis critically regulates synaptic NMDA receptor activity in neuropathic pain. *Cell Rep.* 15 1376–1383. 10.1016/j.celrep.2016.04.039 27160909 PMC4871741

[B107] Li S. An J. Sun C. K. Li Z. W. (2004). Inhibitory effect of caffeine on GABA-activated current in acutely isolated rat dorsal root ganglion neurons. *Sheng Li Xue Bao* 56 384–388. 15224155

[B108] Lidierth M. (2006). Local and diffuse mechanisms of primary afferent depolarization and presynaptic inhibition in the rat spinal cord. *J. Physiol.* 576(Pt 1), 309–327. 10.1113/jphysiol.2006.110577 16873417 PMC1995647

[B109] Lin H. Jun I. Woo J. H. Lee M. G. Kim S. J. Nam J. H. (2019). Temperature-dependent increase in the calcium sensitivity and acceleration of activation of ANO6 chloride channel variants. *Sci. Rep.* 9:6706. 10.1038/s41598-019-43162-1 31040335 PMC6491614

[B110] Liu B. Linley J. E. Du X. Zhang X. Ooi L. Zhang H. (2010). The acute nociceptive signals induced by bradykinin in rat sensory neurons are mediated by inhibition of M-type K+ channels and activation of Ca2+-activated Cl– channels. *J. Clin. Invest.* 120 1240–1252. 10.1172/JCI41084 20335661 PMC2846053

[B111] Liu G. J. Kalous A. Werry E. L. Bennett M. R. (2006). Purine release from spinal cord microglia after elevation of calcium by glutamate. *Mol. Pharmacol.* 70 851–859. 10.1124/mol.105.021436 16760362

[B112] Liu S. Feng J. Luo J. Yang P. Brett T. J. Hu H. (2016). Eact, a small molecule activator of TMEM16A, activates TRPV1 and elicits pain- and itch-related behaviours. *Br. J. Pharmacol.* 173 1208–1218. 10.1111/bph.13420 26756551 PMC4941126

[B113] Liu Y. Zhang H. Men H. Du Y. Xiao Z. Zhang F. (2019). Volume-regulated Cl(-) current: contributions of distinct Cl(-) channels and localized Ca(2+) signals. *Am. J. Physiol. Cell Physiol.* 317 C466–C480. 10.1152/ajpcell.00507.2018 31242393

[B114] Loewen M. E. Forsyth G. W. (2005). Structure and function of CLCA proteins. *Physiol. Rev.* 85 1061–1092. 10.1152/physrev.00016.2004 15987802

[B115] Lucas O. Hilaire C. Delpire E. Scamps F. (2012). KCC3-dependent chloride extrusion in adult sensory neurons. *Mol. Cell. Neurosci.* 50 211–220. 10.1016/j.mcn.2012.05.005 22609694

[B116] Lynch J. W. (2009). Native glycine receptor subtypes and their physiological roles. *Neuropharmacology* 56 303–309. 10.1016/j.neuropharm.2008.07.034 18721822

[B117] Lynch J. W. Zhang Y. Talwar S. Estrada-Mondragon A. (2017). Glycine receptor drug discovery. *Adv. Pharmacol.* 79 225–253. 10.1016/bs.apha.2017.01.003 28528670

[B118] Ma W. Saunders P. A. Somogyi R. Poulter M. O. Barker J. L. (1993). Ontogeny of GABAA receptor subunit mRNAs in rat spinal cord and dorsal root ganglia. *J. Comp. Neurol.* 338 337–359. 10.1002/cne.903380303 7509352

[B119] Maddox F. N. Valeyev A. Y. Poth K. Holohean A. M. Wood P. M. Davidoff R. A. (2004). GABAA receptor subunit mRNA expression in cultured embryonic and adult human dorsal root ganglion neurons. *Brain Res. Dev. Brain Res.* 149 143–151. 10.1016/j.devbrainres.2004.01.001 15063094

[B120] Mair N. Benetti C. Andratsch M. Leitner M. G. Constantin C. E. Camprubi-Robles M. (2011). Genetic evidence for involvement of neuronally expressed S1P(1) receptor in nociceptor sensitization and inflammatory pain. *PLoS One* 6:e17268. 10.1371/journal.pone.0017268 21359147 PMC3040773

[B121] Marcorelles P. Friocourt G. Uguen A. Lede F. Ferec C. Laquerriere A. (2014). Cystic fibrosis transmembrane conductance regulator protein (CFTR) expression in the developing human brain: comparative immunohistochemical study between patients with normal and mutated CFTR. *J. Histochem. Cytochem.* 62 791–801. 10.1369/0022155414546190 25062999

[B122] Mazzone A. Bernard C. E. Strege P. R. Beyder A. Galietta L. J. V. Pasricha P. J. (2011). Altered expression of ano1 variants in human diabetic gastroparesis. *J. Biol. Chem.* 286 13393–13403. 10.1074/jbc.M110.196089 21349842 PMC3075685

[B123] Modol L. Cobianchi S. Navarro X. (2014). Prevention of NKCC1 phosphorylation avoids downregulation of KCC2 in central sensory pathways and reduces neuropathic pain after peripheral nerve injury. *Pain* 155 1577–1590. 10.1016/j.pain.2014.05.004 24813295

[B124] Morales-Aza B. M. Chillingworth N. L. Payne J. A. Donaldson L. F. (2004). Inflammation alters cation chloride cotransporter expression in sensory neurons. *Neurobiol. Dis.* 17 62–69. 10.1016/j.nbd.2004.05.010 15350966

[B125] Mulberg A. E. Resta L. P. Wiedner E. B. Altschuler S. M. Jefferson D. M. Broussard D. L. (1995). Expression and localization of the cystic fibrosis transmembrane conductance regulator mRNA and its protein in rat brain. *J. Clin. Invest.* 96 646–652. 10.1172/jci118080 7542288 PMC185240

[B126] Mulberg A. E. Weyler R. T. Altschuler S. M. Hyde T. M. (1998). Cystic fibrosis transmembrane conductance regulator expression in human hypothalamus. *Neuroreport* 9 141–144. 10.1097/00001756-199801050-00028 9592064

[B127] Mulberg A. E. Wiedner E. B. Bao X. Marshall J. Jefferson D. M. Altschuler S. M. (1994). Cystic fibrosis transmembrane conductance regulator protein expression in brain. *Neuroreport* 5 1684–1688. 7529593 10.1097/00001756-199408150-00035

[B128] Mundhenk L. Alfalah M. Elble R. C. Pauli B. U. Naim H. Y. Gruber A. D. (2006). Both cleavage products of the mCLCA3 protein are secreted soluble proteins. *J. Biol. Chem.* 281 30072–30080. 10.1074/jbc.m606489200 16895902

[B129] Naik A. K. Latham J. R. Obradovic A. Jevtovic-Todorovic V. (2012). Dorsal root ganglion application of muscimol prevents hyperalgesia and stimulates myelin protein expression after sciatic nerve injury in rats. *Anesth. Analg.* 114 674–682. 10.1213/ANE.0b013e31823fad7e 22190549

[B130] Naik A. K. Pathirathna S. Jevtovic-Todorovic V. (2008). GABAA receptor modulation in dorsal root ganglia in vivo affects chronic pain after nerve injury. *Neuroscience* 154 1539–1553. 10.1016/j.neuroscience.2008.04.061 18554816

[B131] Niu N. Zhang J. Guo Y. Yang C. Gu J. (2009). Cystic fibrosis transmembrane conductance regulator expression in human spinal and sympathetic ganglia. *Lab. Invest.* 89 636–644. 10.1038/labinvest.2009.28 19333236

[B132] Obata K. Yamanaka H. Fukuoka T. Yi D. Tokunaga A. Hashimoto N. (2003). Contribution of injured and uninjured dorsal root ganglion neurons to pain behavior and the changes in gene expression following chronic constriction injury of the sciatic nerve in rats. *Pain* 101 65–77. 10.1016/s0304-3959(02)00296-8 12507701

[B133] Obradovic A. L. Scarpa J. Osuru H. P. Weaver J. L. Park J. Y. Pathirathna S. (2015). Silencing the alpha2 subunit of gamma-aminobutyric acid type A receptors in rat dorsal root ganglia reveals its major role in antinociception posttraumatic nerve injury. *Anesthesiology* 123 654–667. 10.1097/ALN.0000000000000767 26164299 PMC4568754

[B134] O’Driscoll K. E. Hatton W. J. Burkin H. R. Leblanc M. Britton F. C. (2008). Expression, localization, and functional properties of Bestrophin 3 channel isolated from mouse heart. *Am. J. Physiol. Cell Physiol.* 295 C1610–C1624. 10.1152/ajpcell.00461.2008 18945938 PMC2603566

[B135] O’Driscoll K. E. Leblanc N. Hatton W. J. Britton F. C. (2009). Functional properties of murine bestrophin 1 channel. *Biochem. Biophys. Res. Commun.* 384 476–481. 10.1016/j.bbrc.2009.05.008 19426717 PMC2705987

[B136] Oh U. Jung J. (2016). Cellular functions of TMEM16/anoctamin. *Pflugers. Arch.* 468 443–453. 10.1007/s00424-016-1790-0 26811235 PMC4751194

[B137] Okada T. Akita T. Sato-Numata K. Islam M. R. Okada Y. (2014). A newly cloned ClC-3 isoform, ClC-3d, as well as ClC-3a mediates Cd-sensitive outwardly rectifying anion currents. *Cell Physiol. Biochem.* 33 539–556. 10.1159/000358633 24603049

[B138] Olsen R. W. (2002). “Chapter 12: GABA,” in *Neuropsychopharmacology – 5th Generation of Progress*, eds Davis K. L. Charney D. Coyle J. T. Nemeroff C. (Philadelphia, PA: Lippincott, Williams & Wilkins).

[B139] Oyelese A. A. Kocsis J. D. (1996). GABAA-receptor-mediated conductance and action potential waveform in cutaneous and muscle afferent neurons of the adult rat: differential expression and response to nerve injury. *J. Neurophysiol.* 76 2383–2392. 10.1152/jn.1996.76.4.2383 8899611 PMC2605353

[B140] Oyelese A. A. Rizzo M. A. Waxman S. G. Kocsis J. D. (1997). Differential effects of NGF and BDNF on axotomy-induced changes in GABA(A)-receptor-mediated conductance and sodium currents in cutaneous afferent neurons. *J. Neurophysiol.* 78 31–42. 10.1152/jn.1997.78.1.31 9242258 PMC2605357

[B141] Padjen A. L. Mitsoglou G. M. Hassessian H. (1989). Further evidence in support of taurine as a mediator of synaptic transmission in the frog spinal cord. *Brain Res.* 488 288–296. 10.1016/0006-8993(89)90720-8 2787189

[B142] Pang R. P. Xie M. X. Yang J. Shen K. F. Chen X. Su Y. X. (2016). Downregulation of ClC-3 in dorsal root ganglia neurons contributes to mechanical hypersensitivity following peripheral nerve injury. *Neuropharmacology* 110(Pt A), 181–189. 10.1016/j.neuropharm.2016.07.023 27460962

[B143] Park H. Han K.-S. Oh S.-J. Jo S. Woo J. Yoon B.-E. (2013). High glutamate permeability and distal localization of Best1 channel in CA1 hippocampal astrocyte. *Mol. Brain* 6:54. 10.1186/1756-6606-6-54 24321245 PMC4029177

[B144] Patel A. C. Brett T. J. Holtzman M. J. (2009). The role of CLCA proteins in inflammatory airway disease. *Annu. Rev. Physiol.* 71 425–449. 10.1146/annurev.physiol.010908.163253 18954282 PMC4017675

[B145] Paulino C. Kalienkova V. Lam A. K. M. Neldner Y. Dutzler R. (2017). Activation mechanism of the calcium-activated chloride channel TMEM16A revealed by cryo-EM. *Nature* 552 421–425. 10.1038/nature24652 29236691

[B146] Payne J. A. Rivera C. Voipio J. Kaila K. (2003). Cation-chloride co-transporters in neuronal communication, development and trauma. *Trends Neurosci.* 26 199–206. 10.1016/s0166-2236(03)00068-7 12689771

[B147] Pfeffer C. K. Stein V. Keating D. J. Maier H. Rinke I. Rudhard Y. (2009). NKCC1-dependent GABAergic excitation drives synaptic network maturation during early hippocampal development. *J. Neurosci.* 29 3419–3430. 10.1523/JNEUROSCI.1377-08.2009 19295148 PMC6665272

[B148] Pieraut S. Lucas O. Sangari S. Sar C. Boudes M. Bouffi C. (2011). An autocrine neuronal interleukin-6 loop mediates chloride accumulation and NKCC1 phosphorylation in axotomized sensory neurons. *J. Neurosci.* 31 13516–13526. 10.1523/JNEUROSCI.3382-11.2011 21940443 PMC6623307

[B149] Pifferi S. Dibattista M. Menini A. (2009). TMEM16B induces chloride currents activated by calcium in mammalian cells. *Pflügers Archiv. Eur. J. Physiol.* 458 1023–1038. 10.1007/s00424-009-0684-9 19475416

[B150] Pineda-Farias J. B. Barragan-Iglesias P. Loeza-Alcocer E. Torres-Lopez J. E. Rocha-Gonzalez H. I. Perez-Severiano F. (2015). Role of anoctamin-1 and bestrophin-1 in spinal nerve ligation-induced neuropathic pain in rats. *Mol. Pain* 11:12990. 10.1186/s12990-015-0042-1 26130088 PMC4487556

[B151] Poet M. Kornak U. Schweizer M. Zdebik A. A. Scheel O. Hoelter S. (2006). Lysosomal storage disease upon disruption of the neuronal chloride transport protein ClC-6. *Proc. Natl. Acad. Sci. U.S.A.* 103 13854–13859. 10.1073/pnas.0606137103 16950870 PMC1564226

[B152] Ponsioen B. van Zeijl L. Langeslag M. Berryman M. Littler D. Jalink K. (2009). Spatiotemporal regulation of chloride intracellular channel protein CLIC4 by RhoA. *Mol. Biol. Cell* 20 4664–4672. 10.1091/mbc.E09-06-0529 19776349 PMC2777097

[B153] Porreca F. Ossipov M. H. Gebhart G. F. (2002). Chronic pain and medullary descending facilitation. *Trends Neurosci.* 25 319–325. 10.1016/s0166-2236(02)02157-4 12086751

[B154] Prabhakar E. Lawson S. N. (1995). The electrophysiological properties of rat primary afferent neurones with carbonic anhydrase activity. *J. Physiol.* 482 609–622. 10.1113/jphysiol.1995.sp020544 7738851 PMC1157786

[B155] Prescott S. A. Sejnowski T. J. Koninck Y. De (2006). Reduction of anion reversal potential subverts the inhibitory control of firing rate in spinal lamina I neurons: towards a biophysical basis for neuropathic pain. *Mol. Pain* 2 1–20. 17040565 10.1186/1744-8069-2-32 PMC1624821

[B156] Price T. J. Cervero F. Gold M. S. Hammond D. L. Prescott S. A. (2009). Chloride regulation in the pain pathway. *Brain Res. Rev.* 60 149–170. 10.1016/j.brainresrev.2008.12.015 19167425 PMC2903433

[B157] Qi Y. Mair N. Kummer K. K. Leitner M. G. Camprubi-Robles M. Langeslag M. (2018). Identification of chloride channels CLCN3 and CLCN5 mediating the excitatory Cl(-) currents activated by sphingosine-1-phosphate in sensory neurons. *Front. Mol. Neurosci.* 11:33. 10.3389/fnmol.2018.00033 29479306 PMC5811518

[B158] Qiu Z. Dubin A. E. Mathur J. Tu B. Reddy K. Miraglia L. J. (2014). SWELL1, a plasma membrane protein, is an essential component of volume-regulated anion channel. *Cell* 157 447–458. 10.1016/j.cell.2014.03.024 24725410 PMC4023864

[B159] Quarta S. Camprubi-Robles M. Schweigreiter R. Matusica D. Haberberger R. V. Proia R. L. (2017). Sphingosine-1-Phosphate and the S1P3 receptor initiate neuronal retraction via RhoA/ROCK associated with CRMP2 phosphorylation. *Front. Mol. Neurosci.* 10:317. 10.3389/fnmol.2017.00317 29066950 PMC5641356

[B160] Raimondo J. V. Markram H. Akerman C. J. (2012). Short-term ionic plasticity at GABAergic synapses. *Front. Synaptic Neurosci.* 4:5 10.3389/fnsyn.2012.00005 PMC3472547 23087642

[B161] Ran R. Gu J. Fu J. Zhong H. Zhao Y. Gu Y. (2014). The role of the GABA-A receptor of the adjacent intact dorsal root ganglion neurons in rats with neuropathic pain. *Acta Neurobiol. Exp.* 74 405–414. 25576971 10.55782/ane-2014-2003

[B162] Reznikov L. R. (2017). Cystic fibrosis and the nervous system. *Chest* 151 1147–1155. 10.1016/j.chest.2016.11.009 27876591 PMC5472519

[B163] Reznikov L. R. Dong Q. Chen J. H. Moninger T. O. Park J. M. Zhang Y. (2013). CFTR-deficient pigs display peripheral nervous system defects at birth. *Proc. Natl. Acad. Sci. U.S.A.* 110 3083–3088. 10.1073/pnas.1222729110 23382208 PMC3581923

[B164] Rivera C. Voipio J. Payne J. A. Ruusuvuori E. Lahtinen H. Lamsa K. (1999). The K+/Cl- co-transporter KCC2 renders GABA hyperpolarizing during neuronal maturation. *Nature* 397 251–255. 10.1038/16697 9930699

[B165] Robertson B. (1989). Characteristics of GABA-activated chloride channels in mammalian dorsal root ganglion neurones. *J. Physiol.* 411 285–300. 10.1113/jphysiol.1989.sp017574 2482355 PMC1190525

[B166] Rocha-Gonzalez H. I. Mao S. Alvarez-Leefmans F. J. (2008). Na+,K+,2Cl- cotransport and intracellular chloride regulation in rat primary sensory neurons: thermodynamic and kinetic aspects. *J. Neurophysiol.* 100 169–184. 10.1152/jn.01007.2007 18385481 PMC2493498

[B167] Rogan M. P. Reznikov L. R. Pezzulo A. A. Gansemer N. D. Samuel M. Prather R. S. (2010). Pigs and humans with cystic fibrosis have reduced insulin-like growth factor 1 (IGF1) levels at birth. *Proc. Natl. Acad. Sci. U.S.A.* 107 20571–20575. 10.1073/pnas.1015281107 21059918 PMC2996661

[B168] Rudomin P. Schmidt R. F. (1999). Presynaptic inhibition in the vertebrate spinal cord revisited. *Exp. Brain Res.* 129 1–37. 10.1007/s002210050933 10550500

[B169] Sabirov R. Z. Merzlyak P. G. Islam M. R. Okada T. Okada Y. (2016). The properties, functions, and pathophysiology of maxi-anion channels. *Pflugers. Arch.* 468 405–420. 10.1007/s00424-015-1774-5 26733413

[B170] Sabirov R. Z. Merzlyak P. G. Okada T. Islam M. R. Uramoto H. Mori T. (2017). The organic anion transporter SLCO2A1 constitutes the core component of the Maxi-Cl channel. *EMBO J.* 36 3309–3324. 10.15252/embj.201796685 29046334 PMC5686547

[B171] Sabirov R. Z. Okada Y. (2005). ATP release via anion channels. *Purinerg. Signal.* 1 311–328. 10.1007/s11302-005-1557-0 18404516 PMC2096548

[B172] Saji M. Obata K. (1981). Stimulus-dependent labeling of cultured ganglionic cell with [14C]2-deoxyglucose. *Brain Res.* 212 435–446. 10.1016/0006-8993(81)90475-3 6112051

[B173] Sala-Rabanal M. Yurtsever Z. Nichols C. G. Brett T. J. (2015). Secreted CLCA1 modulates TMEM16A to activate Ca2+-dependent chloride currents in human cells. *eLife* 4:5875. 10.7554/eLife.05875 25781344 PMC4360653

[B174] Salzer I. Gantumur E. Yousuf A. Boehm S. (2016). Control of sensory neuron excitability by serotonin involves 5HT2C receptors and Ca 2+ -activated chloride channels. *Neuropharmacology* 110 277–286. 10.1016/j.neuropharm.2016.08.006 27511837 PMC6192515

[B175] Schreiber R. Ousingsawat J. Wanitchakool P. Sirianant L. Benedetto R. Reiss K. (2018). Regulation of TMEM16A/ANO1 and TMEM16F/ANO6 ion currents and phospholipid scrambling by Ca(2+) and plasma membrane lipid. *J. Physiol.* 596 217–229. 10.1113/JP275175 29134661 PMC5767690

[B176] Schroeder B. C. Cheng T. Jan Y. N. Jan L. Y. (2008). Expression cloning of TMEM16A as a calcium-activated chloride channel subunit. *Cell* 134 1019–1029. 10.1016/j.cell.2008.09.003 18805094 PMC2651354

[B177] Shypshyna M. S. Veselovs’kyi M. S. (2010). Characteristics of sensory neurotransmission in co-culture of neurons from the dorsal root ganglion and dorsal horn spinal cord in rats. *Fiziol. Zh.* 56 26–36. 10.15407/fz56.04.026 20968035

[B178] Si J. Q. Zhang Z. Q. Li C. X. Wang L. F. Yang Y. L. Li Z. W. (2004). Modulatory effect of substance P on GABA-activated currents from rat dorsal root ganglion. *Acta Pharmacol. Sin.* 25 623–629. 15132829

[B179] Sigel E. Steinmann M. E. (2012). Structure, function, and modulation of GABA(A) receptors. *J. Biol. Chem.* 287 40224–40231. 10.1074/jbc.R112.386664 23038269 PMC3504738

[B180] Sokolova E. Nistri A. Giniatullin R. (2001). Negative cross talk between anionic GABAA and cationic P2X ionotropic receptors of rat dorsal root ganglion neurons. *J. Neurosci.* 21 4958–4968. 10.1523/jneurosci.21-14-04958.2001 11438571 PMC6762830

[B181] Steinmeyer K. Ortland C. Jentsch T. J. (1991). Primary structure and functional expression of a developmentally regulated skeletal muscle chloride channel. *Nature* 354 301–304. 10.1038/354301a0 1659664

[B182] Strege P. R. Bernard C. E. Mazzone A. Linden D. R. Beyder A. Gibbons S. J. (2015). A novel exon in the human Ca2+-activated Cl- channel Ano1 imparts greater sensitivity to intracellular Ca2+. *Am. J. Physiol. Gastrointest. Liver Physiol.* 309 G743–G749. 10.1152/ajpgi.00074.2015 26359375 PMC4628966

[B183] Sun H. Tsunenari T. Yau K.-W. Nathans J. (2002). The vitelliform macular dystrophy protein defines a new family of chloride channels. *Proc. Natl. Acad. Sci. U.S.A.* 99 4008–4013. 10.1073/pnas.052692999 11904445 PMC122639

[B184] Sung K. W. Kirby M. McDonald M. P. Lovinger D. M. Delpire E. (2000). Abnormal GABA(A) receptor-mediated currents in dorsal root ganglion neurons isolated from Na-K-2Cl cotransporter null mice. *J. Neurosci.* 20 7531–7538. 10.1523/jneurosci.20-20-07531.2000 11027211 PMC6772871

[B185] Suzuki M. Mizuno A. (2004). A novel human Cl-channel family related todrosophila flightlesslocus. *J. Biol. Chem.* 279 22461–22468. 10.1074/jbc.m313813200 15010458

[B186] Takayama Y. Uta D. Furue H. Tominaga M. (2015). Pain-enhancing mechanism through interaction between TRPV1 and anoctamin 1 in sensory neurons. *Proc. Natl. Acad. Sci. U.S.A.* 112 5213–5218. 10.1073/pnas.1421507112 25848051 PMC4413337

[B187] Toulme E. Blais D. Leger C. Landry M. Garret M. Seguela P. (2007). An intracellular motif of P2X(3) receptors is required for functional cross-talk with GABA(A) receptors in nociceptive DRG neurons. *J. Neurochem* 102 1357–1368. 10.1111/j.1471-4159.2007.04640.x 17498217

[B188] Valeyev A. Y. Hackman J. C. Holohean A. M. Wood P. M. Katz J. L. Davidoff R. A. (1999). GABA-Induced Cl- current in cultured embryonic human dorsal root ganglion neurons. *J. Neurophysiol.* 82 1–9. 10.1152/jn.1999.82.1.1 10400929

[B189] Valeyev A. Y. Hackman J. C. Wood P. M. Davidoff R. A. (1996). Pharmacologically novel GABA receptor in human dorsal root ganglion neurons. *J. Neurophysiol.* 76 3555–3558. 10.1152/jn.1996.76.5.3555 8930293

[B190] Vikman K. S. Hill R. H. Backstrom E. Robertson B. Kristensson K. (2003). Interferon-gamma induces characteristics of central sensitization in spinal dorsal horn neurons in vitro. *Pain* 106 241–251. 10.1016/s0304-3959(03)00262-8 14659507

[B191] Vlachova V. Lyfenko A. Orkand R. K. Vyklicky L. (2001). The effects of capsaicin and acidity on currents generated by noxious heat in cultured neonatal rat dorsal root ganglion neurones. *J. Physiol.* 533(Pt 3), 717–728. 10.1111/j.1469-7793.2001.t01-1-00717.x 11410629 PMC2278653

[B192] Voss F. K. Ullrich F. Munch J. Lazarow K. Lutter D. Mah N. (2014). Identification of LRRC8 heteromers as an essential component of the volume-regulated anion channel VRAC. *Science* 344 634–638. 10.1126/science.1252826 24790029

[B193] Wang H. C. Cheng K. I. Chen P. R. Tseng K. Y. Kwan A. L. Chang L. L. (2018). Glycine receptors expression in rat spinal cord and dorsal root ganglion in prostaglandin E2 intrathecal injection models. *BMC Neurosci.* 19:72. 10.1186/s12868-018-0470-8 30413143 PMC6230273

[B194] Wang R. Lu Y. Gunasekar S. Zhang Y. Benson C. J. Chapleau M. W. (2017). The volume-regulated anion channel (LRRC8) in nodose neurons is sensitive to acidic pH. *JCI Insight.* 2:e90632. 10.1172/jci.insight.90632 28289711 PMC5333957

[B195] Wanitchakool P. Ousingsawat J. Sirianant L. Cabrita I. Faria D. Schreiber R. (2017). Cellular defects by deletion of ANO10 are due to deregulated local calcium signaling. *Cell. Signal.* 30 41–49. 10.1016/j.cellsig.2016.11.006 27838374

[B196] White G. (1990). GABAA-receptor-activated current in dorsal root ganglion neurons freshly isolated from adult rats. *J. Neurophysiol.* 64 57–63. 10.1152/jn.1990.64.1.57 2167353

[B197] Witschi R. Punnakkal P. Paul J. Walczak J. S. Cervero F. Fritschy J. M. (2011). Presynaptic alpha2-GABAA receptors in primary afferent depolarization and spinal pain control. *J. Neurosci.* 31 8134–8142. 10.1523/JNEUROSCI.6328-10.2011 21632935 PMC3567284

[B198] Wright R. Raimondo J. V. Akerman C. J. (2011). Spatial and temporal dynamics in the ionic driving force for GABA A receptors. *Neural Plast.* 2011:728395. 10.1155/2011/728395 21766044 PMC3135070

[B199] Wu X. P. Li Z. W. Fan Y. Z. (1994). Inhibitory effect of SP on GABA-activated currents in freshly isolated rat DRG neurons. *Sheng Li Xue Bao* 46 586–590. 7533331

[B200] Xi Z. X. Akasu T. (1996). N-methyl-D-aspartate depresses GABAA receptor-mediated currents in neurons of bullfrog dorsal root ganglia. *Neurosci. Lett.* 212 17–20. 10.1016/0304-3940(96)12771-3 8823752

[B201] Xiong W. Cui T. Cheng K. Yang F. Chen S. R. Willenbring D. (2012). Cannabinoids suppress inflammatory and neuropathic pain by targeting alpha3 glycine receptors. *J. Exp. Med.* 209 1121–1134. 10.1084/jem.20120242 22585736 PMC3371734

[B202] Yamada K. Akasu T. (1996). Substance P suppresses GABAA receptor function via protein kinase C in primary sensory neurones of bullfrogs. *J. Physiol.* 496(Pt 2), 439–449. 10.1113/jphysiol.1996.sp021697 8910228 PMC1160889

[B203] Yang T. Liu Q. Kloss B. Bruni R. Kalathur R. C. Guo Y. (2014). Structure and selectivity in bestrophin ion channels. *Science* 346 355–359. 10.1126/science.1259723 25324390 PMC4341822

[B204] Yang Y. D. Cho H. Koo J. Y. Tak M. H. Cho-Shim Y. W. Park S. (2008). TMEM16A confers receptor-activated calcium-dependent chloride conductance. *Nature* 455 1210–1215. 10.1038/nature07313 18724360

[B205] Yang Y. L. Yao K. H. Gu Y. Z. Guan B. C. Li Z. W. (2003). Three kinds of current in response to substance P in bullfrog DRG neurons. *Brain Res.* 981 70–77. 10.1016/s0006-8993(03)02949-4 12885427

[B206] Ye W. Han T. W. He M. Jan Y. N. Jan L. Y. (2019). Dynamic change of electrostatic field in TMEM16F permeation pathway shifts its ion selectivity. *eLife* 8:e45187. 10.7554/eLife.45187 31318330 PMC6690719

[B207] Zeilhofer H. U. Mohler H. Di Lio A. (2009). GABAergic analgesia: new insights from mutant mice and subtype-selective agonists. *Trends Pharmacol. Sci.* 30 397–402. 10.1016/j.tips.2009.05.007 19616317

[B208] Zhang M. Gao C. X. Wang Y. P. Ma K. T. Li L. Yin J. W. (2018). The association between the expression of PAR2 and TMEM16A and neuropathic pain. *Mol. Med. Rep.* 17 3744–3750. 10.3892/mmr.2017.8295 29257338 PMC5802179

[B209] Zhang Y. Chen K. Sloan S. A. Bennett M. L. Scholze A. R. O’Keeffe S. (2014). An RNA-sequencing transcriptome and splicing database of glia, neurons, and vascular cells of the cerebral cortex. *J. Neurosci.* 34 11929–11947. 10.1523/JNEUROSCI.1860-14.2014 25186741 PMC4152602

[B210] Zhang Y. Zhao S. Rodriguez E. Takatoh J. Han B. X. Zhou X. (2015). Identifying local and descending inputs for primary sensory neurons. *J. Clin. Invest.* 125 3782–3794. 10.1172/JCI81156 26426077 PMC4607134

[B211] Zhang Y. H. Fehrenbacher J. C. Vasko M. R. Nicol G. D. (2006). Sphingosine-1-phosphate via activation of a G-protein-coupled receptor(s) enhances the excitability of rat sensory neurons. *J. Neurophysiol.* 96 1042–1052. 10.1152/jn.00120.2006 16723416

[B212] Zhu Y. Lu S. G. Gold M. S. (2012). Persistent inflammation increases GABA-induced depolarization of rat cutaneous dorsal root ganglion neurons in vitro. *Neuroscience* 220 330–340. 10.1016/j.neuroscience.2012.06.025 22728089 PMC3412885

[B213] Zimmerman A. L. Kovatsis E. M. Pozsgai R. Y. Tasnim A. Zhang Q. Ginty D. D. (2019). Distinct modes of presynaptic inhibition of cutaneous afferents and their functions in behavior. *Neuron* 102 420–434.e8. 10.1016/j.neuron.2019.02.002 30826183 PMC6472967

